# Sex and All-Cause Mortality in the US, 1999 to 2019

**DOI:** 10.1001/jamanetworkopen.2025.56299

**Published:** 2026-01-30

**Authors:** Julia Francis, Barry I. Graubard, Hormuzd Katki, Sarah S. Jackson

**Affiliations:** 1 Division of Cancer Epidemiology and Genetics, National Cancer Institute, National Institutes of Health, Rockville, Maryland

## Abstract

**Importance:**

While life expectancy in the US has increased among both sexes, a significant sex gap in mortality has been consistently observed during the past 2 decades.

**Objective:**

To examine differences in mortality rates between males and females for overall mortality and the leading 9 causes of mortality (coded by the National Death Index as diseases of the heart, malignant neoplasms, chronic lower respiratory diseases, unintentional injuries, cerebrovascular diseases, Alzheimer disease, diabetes, influenza and pneumonia, and nephritis, nephrotic syndrome, and nephrosis).

**Design, Setting, and Participants:**

This prospective cohort study included adults 20 years or older participating in the National Health and Nutrition Examination Survey (NHANES) cycles between 1999 and 2016. Data were linked to the National Death Index from the date of survey participation through December 31, 2019. Data were analyzed from July 16, 2024, to August 14, 2025.

**Exposure:**

Participation in the 1999-2016 NHANES cycles.

**Main Outcomes and Measures:**

Cox proportional hazards regression was used to estimate male-to-female hazard ratios (MF HR) for overall and cause-specific mortality, adjusting for sociodemographic characteristics (eg, age, race and ethnicity), behavioral factors (eg, smoking, alcohol use), and chronic conditions (eg, diabetes, hypertension). The adjusted MF HRs were stratified by self-rated health, race and ethnicity, income, and educational level.

**Results:**

Among 47 056 participants (52.0% female and 48.0% male [weighted]), 12.9% (12.2% females and 13.6% males) were deceased at the end of follow-up. Males had a 63% higher risk of all-cause mortality compared with females, with the largest difference in mortality among individuals who died from heart disease (MF HR, 1.96; 95% CI, 1.72-2.24). The MF HR differed significantly across race and ethnicity for heart disease (range, 0.92 [95% CI, 0.36-2.35] for individuals of other race or ethnicity to 2.11 [95% CI, 1.80-2.48] for White individuals; *P* = .02 for interaction) and across income quartiles for mortality due to cerebrovascular diseases (range, 0.59 [95% CI, 0.25-1.38] for highest to 2.25 [95% CI, 1.31-3.86] for lowest income; *P* = .03 for interaction) and accidents (range, 0.57 [95% CI, 0.16-1.99] for highest income to 2.40 [95% CI, 1.03-5.58] for third highest income; *P* = .02 for interaction). No difference in the MF HR was found across self-rated health categories or education attainment levels. Information on covariates was largely collected by self-report, resulting in underreporting or overreporting due to social desirability bias. How these factors may have changed leading up to the time of mortality could not be accounted for.

**Conclusions:**

In this cohort study of adult NHANES participants, after accounting for a wide range of risk factors, the sex gap in mortality remained for most causes of mortality, suggesting there may be intrinsic biological factors (eg, sex hormones, chromosomes, immune response) associated with sex differences in mortality. Further research should investigate the effects of sex-linked biological factors on mortality.

## Key Points

**Question:**

Are there differences in mortality rates between males and females for all-cause mortality and the leading 9 causes of mortality?

**Findings:**

In this cohort study of 47 056 adults participating in the National Health and Nutrition Examination Survey, after accounting for demographic characteristics (eg, age, race and ethnicity), behavioral factors (eg, smoking, alcohol use), and chronic conditions (eg, diabetes, hypertension), males had a 63% greater risk of all-cause mortality than females.

**Meaning:**

These findings suggest that there may be intrinsic biological factors (sex hormones, chromosomes, or immune response) associated with sex differences in mortality.

## Teaser

This cohort study uses data from the National Health and Nutrition Examination Survey (NHANES) to compare mortality rates between male and female individuals in the US for overall mortality and the 9 leading causes of death.

## Introduction

Although life expectancy for both sexes has increased steadily in the US during the 20th and 21st centuries, the sex gap in mortality has remained.^1,2^ In 2023, the life expectancy was 75.8 years for males and 81.8 years for females.^3^ Mortality-related statistics also show that the leading causes of mortality differ by sex. The 5 leading causes of mortality for males were heart disease (23%), cancer (20%), unintentional injuries (9%), stroke (4%), and chronic lower respiratory disease (4%), whereas the leading causes for females were heart disease (21%), cancer (20%), stroke (6%), Alzheimer disease (5%), and chronic lower respiratory disease (5%).^3^ These differences suggest the possibility of biological factors (eg, sex hormones, chromosome complement), in addition to risk factors related to sociodemographic characteristics, behavior, and chronic conditions influencing these sex differences in mortality.^4^

In high income countries, risk factors for mortality in addition to sex include age, race, ethnicity, educational level, income, access to health care, alcohol use, and smoking habits.^5,6,7,8^ Differential exposure to some, or all, of these factors may influence the incidence of cause-specific mortality in males and females.^1,9^ For example, males may have a higher prevalence of harmful health behaviors, such as smoking and alcohol use, compared with females, who have a higher prevalence of health-seeking behaviors.^10^ There may also be biological factors influencing the mortality rate in males and females. Differences in sex hormone levels, chromosomal characteristics, and immune response between males and females may contribute to this sex gap in mortality.^11,12^

Few studies have compared male and female mortality rates in the general population adjusting for behavioral and lifestyle differences to account for differential prevalence of these factors between the sexes. A previous study^13^ found that males had higher age-adjusted mortality rates than females and indicated that these differences may be due to differences in health behaviors, chronic conditions, and socioeconomic factors, but the investigators could not account for the factors directly. Other studies that have presented sex-stratified mortality rates focusing on the sex gap within specific chronic conditions (such as chronic kidney disease and chronic obstructive pulmonary disease) found that males had a higher risk of all-cause mortality.^10,14,15^ Adjusting for sociodemographic characteristics, behaviors, and chronic conditions in all-cause and cause-specific mortality estimates can aid in our understanding of how much intrinsic biological differences, above and beyond these external factors, might contribute to mortality. An analysis by Rogers et al^1^ using population-based data from 1988 to 1995 found that the sex gap remained after adjusting for mortality risk factors and concluded that with improved health behaviors among males, the sex gap would narrow. We sought to investigate this assertion using more recent data from a nationally representative sample to examine sex differences in all-cause mortality and for the top 9 causes of mortality, adjusting for sociodemographic characteristics, comorbid conditions, lifestyle factors, and access to health care. We also explored the association between sex and mortality across self-rated health categories, racial and ethnic groups, levels of education, and income.

## Methods

### Data Source, Data Collection, and Covariates

This cohort study followed the Strengthening the Reporting of Observational Studies in Epidemiology (STROBE) reporting guideline. The National Center for Health Statistics Ethics Review Board have approved each National Health and Nutrition Examination Survey (NHANES) study protocol since the survey began running continuously in 1999. Written informed consent was obtained from all participants.

NHANES collected nationally representative data on a wide range of demographic characteristics, lifestyle and behavioral factors, and health conditions in the US population.^16^ We used publicly available NHANES data collected between 1999 and 2016, linked by the National Center for Health Statistics to death records from National Death Index (NDI) from 1999 to 2019.^17^ These public-use linked mortality files provide mortality follow-up data from the date of survey participation through December 31, 2019.^17^ The mortality files contain coding for all-cause mortality and the leading 9 causes of mortality using the *International Statistical Classification of Diseases, Tenth Revision*, injuries, and causes of death guidelines.^17^ The available causes of mortality as coded in the NDI included diseases of the heart, malignant neoplasms, chronic lower respiratory diseases, accidents (unintentional injuries), cerebrovascular diseases, Alzheimer disease, diabetes, influenza and pneumonia, and nephritis, nephrotic syndrome, and nephrosis (eTable 1 in Supplement 1). The data files from NHANES and the NDI were downloaded on July 16, 2024.

We extracted and harmonized variables from the NHANES interviewer-administered questionnaire across all cycles to weigh the respondents back to the population (eTable 2 in Supplement 1). The demographic variables included sex (male or female), age (continuous in years), self-reported race and ethnicity (Hispanic, non-Hispanic Black [Black], non-Hispanic White [White], or other race or ethnicity [including non-Hispanic Asian American and multiracial]), educational level (high school or less, some college or associate degree, or college graduate and above), marital status (married or living with partner; widowed, separated, or divorced; or never married), health insurance coverage (yes or no), and occupational exposure (low physical activity, high physical activity, hazardous high physical activity, missing, or unspecified occupation). We categorized the poverty-income ratio (PIR), which is the ratio of family income to poverty guidelines specific to the survey year, into quartiles, where the highest PIR quartile represents the greatest wealth. For the lifestyle variables, we categorized the number of alcoholic drinks per day (little to no drinking: ≤1 drink; moderate drinking: >1 drink but ≤2 drinks; heavy drinking: >2 drinks [individuals with values indicating >12 drinks per day were excluded]), smoking intensity (nonsmoker; past smoker: ≤10 cigarettes/d; past smoker: >10 cigarettes/d; current smoker: ≤10 cigarettes/d; current smoker: >10 cigarettes/d), physical activity defined as at least 10 minutes of exercise causing sweat (yes or no), and sedentary behavior (no television or computer use, ≤2 h/d, 3-4 h/d, or ≥5 h/d). For the health-related variables, we categorized self-rated health (excellent, very good, good, fair or poor [combined due to the small number of participants who responded with poor]), history of cardiovascular disease (CVD) (yes or no), history of diabetes (yes or no), history of hypertension (high blood pressure; yes or no), and history of cancer (yes or no). Body mass index (calculated as weight in kilograms divided by height in meters squared) was categorized using the Centers for Disease Control and Prevention guidance for adults (underweight, <18.5; normal weight, ≥18.5-25.0; overweight, ≥25.0; or obesity, ≥30.0)^18^ collected on physical examination.

### Study Population

The 1999-2016 NHANES sample consisted of 92 062 noninstitutionalized individuals of all ages. We excluded 42 550 participants younger than 20 years and 91 additional participants who were unable to be linked to the NDI. Individuals who completed the NHANES questionnaire but did not participate in the mobile examination center portion of the interview were also excluded (2365 participants) resulting in a total of 47 056 participants (48.0% males and 52.0% females) used in the present analysis.

### Statistical Analyses

NHANES has a complex stratified multistage cluster sample design with a sample weight for each of the randomly selected study participants, reflecting the number of individuals in the US that the participant represents. All analyses account for the complex sample design in the estimation, 95% CIs, and statistical testing. Data were analyzed from July 16, 2024, to August 14, 2025. We conducted a descriptive analysis to summarize health and demographic data of the sample. We calculated weighted mortality rates for males and females, separately, for all-cause and cause-specific mortality. To estimate the risk of all-cause mortality and cause-specific mortality in males compared with females, we used Cox proportional hazards regression to calculate the male-to-female hazard ratios (MF HRs) and 95% CIs. Person-years were calculated from the age at NHANES interview to the age at death or end of NDI coverage. All models were adjusted for age, age^2^ (to account for nonlinear associations), race and ethnicity, educational level, PIR, health insurance coverage, marital status, routine place to go for health care, occupation, smoking intensity, alcohol use, and body mass index. We additionally adjusted for sedentary behavior, physical activity, history of CVD, history of hypertension, history of diabetes, and history of cancer based on cause of mortality (eTable 3 in Supplement 1). We examined whether MF HRs for each cause of mortality differed across race and ethnicity, income, self-rated health, and educational level with an interaction term in the model.

We estimated the all-cause MF HR in Hispanic individuals and individuals who identified as other race stratified by whether participants were born in or outside the US, although we found no difference in HRs between these 2 groups. We also tested the proportional hazard assumption including a sex and person-time interaction into the model. All the models met the proportional hazard assumption.

All analyses were conducted using SAS-callable SUDAAN software, release 11.0.4 (SAS Institute Inc). All *P* values were 2 sided without correction for multiple comparisons, with *P* < .05 indicating statistical significance. Complete case analysis was used for missing data.

## Results

### Participant Characteristics

A total of 47 056 participants, representing 214 497 593 individuals (48.0% males and 52.0% females) were eligible for analysis, of whom 12.9% had died (13.6% males and 12.2% females) (Table 1). Participants contributed a total of 539 748 person-years with a mean of 10 person-years per male participant and 11 person-years per female participant. The mean age of male participants was 46.1 (IQR, 31.8- 7.5) years, while the mean age of female participants was 47.6 (IQR, 32.8-59.8) years at the time of the NHANES interview. Black individuals comprised 11.3% of the sample (12.0% of females and 10.4% of males); Hispanic, 13.6% of the sample (13.1% females and 14.1% males); White, 68.7% of the sample (68.3% of females and 69.1% of males); and other race, 6.5% of the sample (6.6% of females and 6.4% of males). Among male participants, 27.9% graduated from college compared with 26.6% of female participants. Nonsmokers made up 53.3% of the sample (61.4% of females and 47.8% of males). Furthermore, 47.1% of males and 66.3% of females had 1 or fewer alcoholic drinks per day. The highest quartile for PIR included 23.2% of males and 27.1% of females. For self-rated health, 18.2% of male and 17.0% female participants perceived themselves to be in excellent health. The most common causes of mortality for both sexes were heart disease and malignant neoplasms.

**Table 1.** Demographic, Health, Lifestyle, and Mortality Characteristics of NHANES Study Participants, 1999 to 2019

Characteristic	No. (weighted %) of participants		
	Total	Males	Females
Total participants eligible	47 056 (100)	22 624 (48.0)	24 432 (52.0)
Total eligible participants assumed alive	38 874 (87.1)	18 112 (86.4)	20 762 (87.8)
Total eligible participants deceased	8182 (12.9)	4512 (13.6)	3670 (12.2)
**Deaths by survey years**			
1999-2000	1441 (24.1)	773 (25.2)	668 (23.0)
2001-2002	1385 (21.1)	755 (22.0)	630 (20.3)
2003-2004	1274 (17.7)	689 (19.0)	585 (16.5)
2005-2006	950 (14.9)	523 (15.1)	427 (14.6)
2007-2008	1047 (12.8)	588 (13.6)	459 (12.1)
2009-2010	813 (10.1)	472 (11.2)	341 (9.1)
2011-2012	578 (8.5)	326 (9.6)	252 (7.4)
2013-2014	444 (6.6)	226 (6.5)	218 (6.7)
2015-2016	250 (3.4)	160 (3.9)	90 (2.9)
**Demographic **			
Age range, y			
20-29	8347 (18.8)	3829 (19.6)	4518 (18.1)
30-39	8031 (18.8)	3795 (19.3)	4236 (18.4)
40-49	7974 (20.1)	3793 (20.4)	4181 (19.8)
50-59	6918 (17.7)	3433 (18.0)	3485 (17.4)
60-69	7442 (12.3)	3672 (12.1)	3770 (12.5)
70-79	5028 (7.8)	2553 (7.1)	2475 (8.4)
≥80	3316 (4.4)	1549 (3.4)	1767 (5.3)
Race and ethnicity			
Hispanic	12 248 (13.6)	5724 (14.1)	6524 (13.1)
Non-Hispanic Black	9816 (11.3)	4694 (10.4)	5122 (12.0)
Non-Hispanic White	21 173 (68.7)	10 363 (69.1)	10 810 (68.3)
Other^a^	3819 (6.5)	1843 (6.4)	1976 (6.6)
Educational level			
High school or less	24 083 (42.1)	11 943 (43.3)	12 140 (40.9)
Some college or associate degree	13 016 (30.7)	5777 (28.8)	7239 (32.5)
College graduate or above	9886 (27.2)	4873 (27.9)	5013 (26.6)
No. missing	71	31	40
Family income to poverty ratio			
First quantile (lowest income)	15 228 (24.8)	6882 (22.3)	8346 (27.2)
Second quantile	11 527 (25.2)	5628 (24.8)	5899 (25.4)
Third quantile	8725 (24.9)	4334 (25.7)	4391 (24.2)
Fourth quantile (highest income)	7546 (25.1)	3904 (27.1)	3642 (23.2)
No. missing	4030	1876	2154
Marital status			
Married or living with partner	27 994 (63.5)	14 724 (67.4)	13 270 (60.0)
Widowed, separated, or divorced	10 396 (18.7)	3518 (12.7)	6878 (24.2)
Never married	8161 (17.8)	4160 (19.9)	4001 (15.8)
No. missing	505	222	283
Occupation			
Nonhazardous, low physical activity	13 245 (35.7)	6027 (35.1)	7218 (36.2)
Nonhazardous, high physical activity	4337 (9.9)	1397 (6.6)	2940 (12.9)
Hazardous, high physical activity	7969 (17.1)	6202 (28.7)	1767 (6.4)
Missing or unspecified	21 505 (37.3)	8998 (29.5)	12 507 (44.5)
**Health and lifestyle **			
BMI			
Underweight (<18.5)	755 (1.7)	283 (1.1)	472 (2.3)
Normal (≥18.5 to <24.9)	13 185 (30.2)	6139 (27.0)	7046 (33.2)
Overweight (≥25 to <29.9)	15 646 (33.6)	8671 (39.3)	6975 (28.2)
Obesity (≥30.0)	16 496 (34.5)	7054 (32.6)	9442 (36.3)
No. missing	974	477	497
Self-reported general health			
Excellent	7032 (17.6)	3537 (18.2)	3495 (17.0)
Very good	12 213 (31.3)	5893 (31.5)	6320 (31.1)
Good	16 440 (33.4)	7948 (33.5)	8492 (33.3)
Fair or poor	11 336 (17.7)	5227 (16.7)	6109 (18.6)
No. missing	35	19	16
Health insurance coverage			
Yes	36 783 (81.9)	17 145 (79.3)	19 638 (84.3)
No	10 053 (18.1)	5375 (20.7)	4678 (15.7)
No. missing	220	104	116
Routine place to go for health care			
Yes	39 755 (85.0)	17 984 (79.4)	21 771 (90.1)
No	7298 (15.0)	4638 (20.6)	2660 (9.9)
No. missing	3	2	1
No. of alcoholic drinks consumed/d			
Little to none (≤1 drink)	28 913 (57.1)	11 636 (47.1)	17 277 (66.3)
Moderate (>1 to ≤2 drinks)	7623 (19.1)	3852 (19.7)	3771 (18.6)
Heavy (>2 drinks)	10 242 (23.8)	6900 (33.2)	3342 (15.1)
No. missing	278	236	42
Smoking intensity			
Nonsmoker	25 396 (53.3)	9930 (47.8)	15 466 (61.4)
Past, ≤10 cigarettes/d	6235 (12.8)	3320 (13.0)	3005 (12.7)
Past, >10 cigarettes/d	5401 (11.8)	3732 (15.8)	1669 (8.2)
Current, ≤10 cigarettes/d	9160 (19.7)	5200 (22.1)	3960 (17.5)
Current, >10 cigarettes/d	813 (2.4%)	506 (3.0%)	307 (1.8%)
No. missing	51	26	25
Physical activity (10 min of exercise causing sweat)			
Yes	19 805 (48.3)	9616 (48.4)	10 189 (48.2)
No or unable to exercise	27 237 (51.7)	13 002 (51.6)	14 235 (51.8)
No. missing	14	6	8
Sedentary behavior, television and/or computer use, h/d			
None to <2	10 401 (18.1)	4997 (17.9)	5404 (18.2)
≥2	21 588 (51.0)	10 227 (50.9)	11 361 (51.2
3-4	7306 (15.0)	3669 (15.5)	3637 (14.5)
≥5	7706 (15.9)	3705 (15.7)	4002 (16.1)
No. missing	54	26	28
Self-reported history of CVD			
Yes	1942 (3.4)	1288 (4.5)	654 (2.4)
No	44 892 (96.6)	21 208 (95.5)	23 684 (97.6)
No. missing	222	128	94
Self-reported history of diabetes			
Yes	5524 (8.6)	2770 (8.8)	2754 (8.4)
No/borderline	41 501 (91.4)	19 840 (91.2)	21 661 (91.6)
No. missing	31	14	17
Self-reported history of hypertension			
Yes	16 126 (30.3)	7610 (29.4)	8516 (31.2)
No	30 729 (69.7)	14 878 (70.6)	15 851 (68.8)
No. missing	201	136	65
Self-reported history of cancer			
Yes	4312 (9.4)	2043 (8.3)	2269 (10.4)
No	242 694 (90.6)	20 560 (91.7)	22 134 (89.6)
No. missing	50	21	29

Abbreviations: BMI, body mass index (calculated as weight in kilograms divided by height in meters squared); CVD, cardiovascular disease.

^a^ Includes Asian American and multiracial.

### Sex and Mortality

Table 2 presents the weighted mortality rates per 100 000 person-years for males and females and the age-adjusted and the fully adjusted MF HRs for overall mortality and the top 9 causes of mortality. The most common cause of mortality for males was malignant neoplasms (weighted mortality rate, 330.6 [95% CI, 301.5-359.6] per 100 000 person-years for males); for females, the most common cause was heart disease (weighted mortality rate, 268.9 [95% CI, 242.8-295.0] per 100 000 person-years for females). For all-cause mortality, males had a 63% higher risk of mortality than females after adjustment for sociodemographic, lifestyle factors, comorbid conditions, and access to health care (MF HR, 1.63; 95% CI, 1.53-1.75). The 3 causes of mortality with the highest adjusted MF HRs were heart disease (MF HR, 1.96; 95% CI, 1.72-2.24), diabetes (MF HR, 1.90; 95% CI, 1.31-2.75), and cerebrovascular diseases (MF HR, 1.72; 95% CI, 1.23-2.41).

**Table 2.** Weighted Mortality Rates for Males and Females With Age-Adjusted and Fully Adjusted Male-to-Female Hazard Ratios for All-Cause and Top 9 Causes of Mortality

Cause of mortality	Weighted mortality rate per 100 000 person-years		MF HR (95% CI)	
	Males	Females	Adjusted for age^a^	Fully adjusted^b^
All-cause	1267.9 (1209.5-1326.3)	1116.9 (1061.4-1172.4)	1.47 (1.40-1.54)	1.63 (1.53-1.75)^c^
Malignant neoplasms	330.6 (301.5-359.6)	227.0 (204.9-249.0)	1.75 (1.56-1.97)	1.68 (1.46-1.94)^d^
Diseases of the heart	316.5 (290.7-342.2)	268.9 (242.8-295.0)	1.60 (1.45-1.77)	1.96 (1.72-2.24)^e^
Chronic lower respiratory diseases	69.7 (57.5-81.8)	77.9 (66.0-89.9)	1.17 (0.93-1.46)	0.96 (0.74-1.25)^f^
Cerebrovascular diseases	62.7 (52.3-73.1)	59.4 (49.1-69.7)	1.46 (1.15-1.87)	1.72 (1.23-2.41)^g^
Unintentional injuries	53.7 (42.9-64.6)	40.6 (31.6-49.7)	1.44 (1.05-1.97)	1.50 (1.06-2.12)^f^
Diabetes	43.9 (34.9-52.9)	39.3 (30.9-47.6)	1.34 (1.03-1.75)	1.90 (1.31-2.75)^h^
Alzheimer disease	31.7 (25.4-38.1)	55.1 (46.4-63.9)	0.92 (0.71-1.19)	1.12 (0.79-1.63)^i^
Nephritis, nephrotic syndrome, and nephrosis	24.1 (17.6-30.5)	25.1 (19.2-31.0)	1.27 (0.90-1.78)	1.48 (0.94-2.32)^j^
Influenza and pneumonia	20.1 (14.7-25.5)	24.8 (17.5-32.0)	1.17 (0.79-1.74)	1.53 (0.92-2.57)^g^

Abbreviation: MF HR, male-to-female hazard ratio.

^a^ Adjusted for age and age^2^.

^b^ Adjusted for age, age^2^, race and ethnicity, educational level, family income-to-poverty ratio, health insurance coverage, marital status, routine place to go for health care, occupation, smoking intensity, alcohol use, and body mass index.

^c^ Additionally adjusted for sedentary behavior, physical activity, and history of cardiovascular disease (CVD), hypertension, diabetes, and cancer.

^d^ Additionally adjusted for physical activity and history of cancer.

^e^ Additionally adjusted for sedentary behavior, physical activity, and history of CVD and hypertension.

^f^ Additionally adjusted for sedentary behavior.

^g^ Additionally adjusted for sedentary behavior and history of CVD and hypertension.

^h^ Additionally adjusted for sedentary behavior, physical activity, and history of hypertension, diabetes, and cancer.

^i^ Additionally adjusted for physical activity.

^j^ Additionally adjusted for physical activity and history of CVD, hypertension, and diabetes.

### Race and Ethnicity

**Figure 1.**
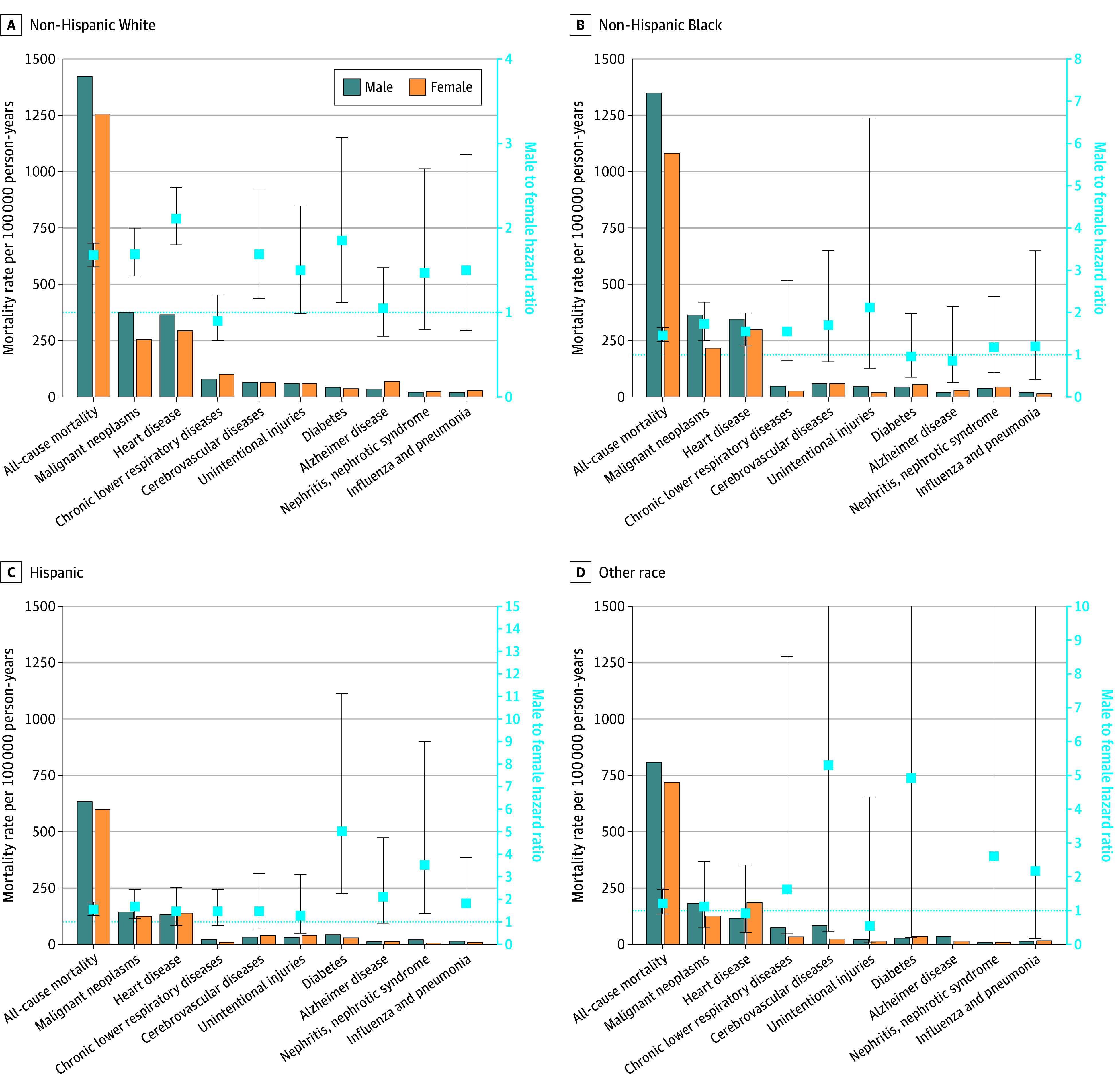
Weighted Male and Female Mortality Rates and Adjusted Male-to-Female Hazard Ratios for All-Cause and the Top 9 Causes of Mortality by Race and Ethnicity Data are ordered by highest to lowest male mortality rates. Bars indicate mortality rates; boxes, male-to-female hazard ratios; whiskers, 95% CIs; and dotted line, the line of unity. The male-to-female hazard ratio estimate for Alzheimer disease among Hispanic individuals is not shown on the graph as the estimate is outside the axis limits.

Across all racial and ethnic groups, males had higher all-cause mortality rates than females for most mortality causes (Figure 1 and eTable 4 in Supplement 1). Among White individuals, all-cause mortality rates were 1424.0 (95% CI, 1348.2-1499.7) per 100 000 person-years for males and 1256.4 (95% CI, 1183.2-1329.6) per 100 000 person-years for females. Among Black individuals, all-cause mortality rates were 1350.3 (95% CI, 1236.4-1464.1) per 100 000 person-years for males and 1082.6 (95% CI, 999.9-1165.3) per 100 000 person-years for females. Among Hispanic individuals, all-cause mortality rates were 635.4 (95% CI, 533.2-737.6) per 100 000 person-years for males and 601.1 (95% CI, 459.3-742.9) per 100 000 person-years for females. Among individuals of other race or ethnicity, all-cause mortality rates were 809.5 (95% CI, 643.0-975.9) per 100 000 person-years for males and 720.3 (95% CI, 578.0-862.7) per 100 000 person-years for females. Across all races and ethnicities, the most common cause of mortality for males was malignant neoplasms and for females was heart disease. Diseases of the heart constituted the only cause of mortality for which the sex gap differed by race and ethnicity after adjustment. Here the sex difference was greatest for White individuals and smallest for individuals of other race or ethnicity (Black MF HR, 1.55 [95% CI, 1.21- 1.99]; Hispanic MF HR, 1.47 [95% CI, 0.85-2.54]; White MF HR, 2.11 [95% CI, 1.80-2.48]; other race or ethnicity MF HR, 0.92 [95% CI, 0.36-2.35]; *P* = .02 for interaction) (Figure 1 and eTable 5 in Supplement 1). The test for interaction found no difference in other MF HRs across race and ethnicity for specific causes of mortality. White males had a consistently higher risk of mortality compared with females from malignant neoplasms, diseases of the heart, cerebrovascular diseases, and diabetes; Black males had a higher risk of mortality from malignant neoplasms and diseases of the heart; Hispanic males had higher mortality from malignant neoplasms, diabetes, and nephritis. No sex differences were seen for any mortality cause for individuals of other race or ethnicity.

### Income-to-Poverty Ratio

**Figure 2.**
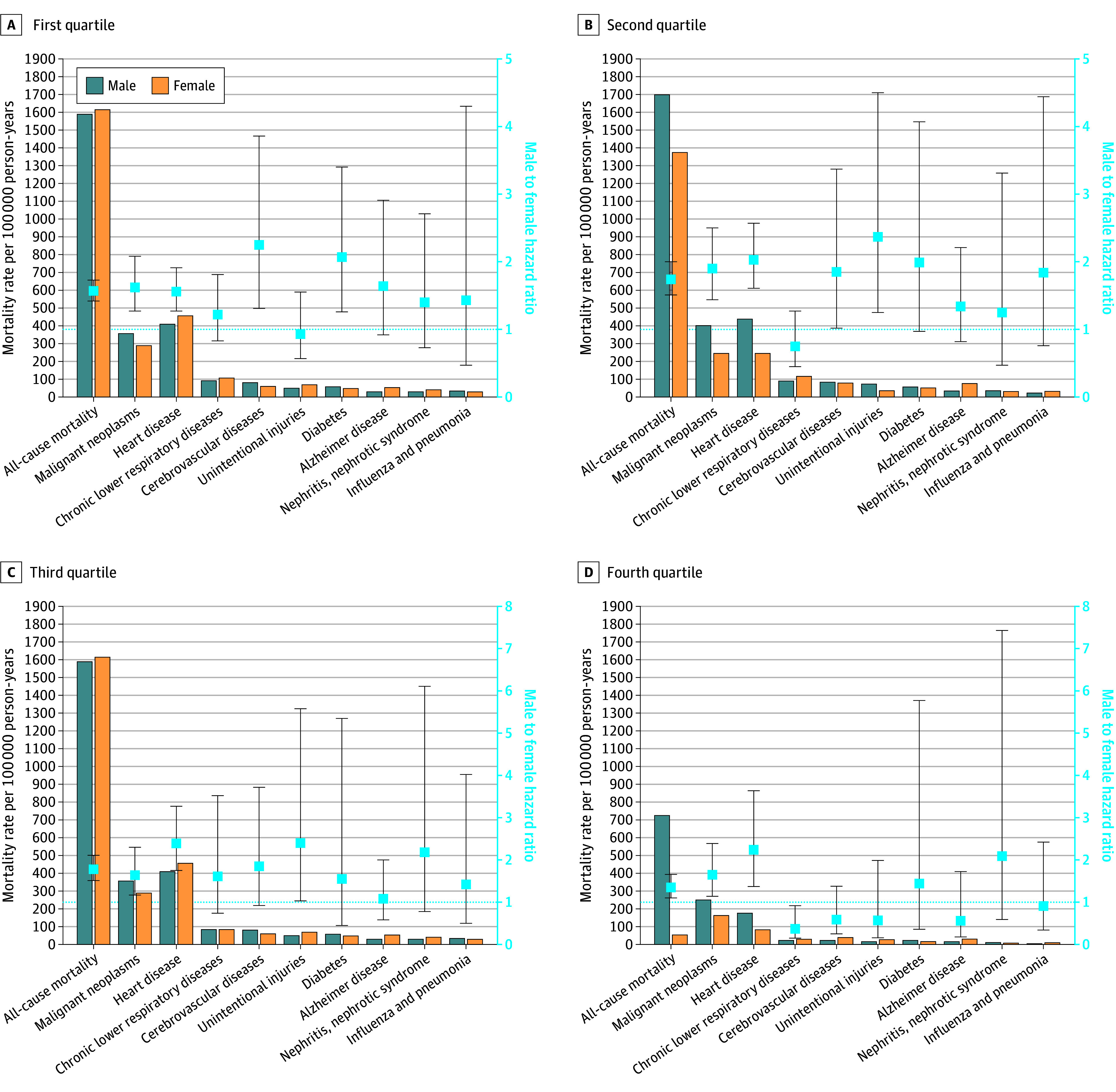
Weighted Male and Female Mortality Rates and Adjusted Male-to-Female Hazard Ratios for All-Cause and the Top 9 Causes of Mortality by Family Income-to-Poverty Ratio Quartiles Data are ordered by highest to lowest male mortality rates. Bars indicate mortality rates; boxes, male-to-female hazard ratios; whiskers, 95% CIs; and dotted line, the line of unity.

All-cause mortality decreased with increasing economic status as measured by the PIR (first quartile: 1590.6 [95% CI, 1477.9-1703.4] per 100 000 person-years for males and 1615.9 [95% CI, 1502.1-1729.7] per 100 000 person-years for females; second quartile: 1700.3 [95% CI, 1575.5-1825.1] per 100 000 person-years for males and 1375.6 [95% CI, 1271.9-1479.4] per 100 000 person-years for females; third quartile: 1089.3 [95% CI, 997.0-1,181.6] per 100 000 person-years for males and 750.8 [95% CI, 662.9-838.7] per 100 000 person-years for females; fourth quartile: 726.4 [95% CI, 646.8-806.0] per 100 000 person-years for males and 555.2 [95% CI, 487.1-623.4] per 100 000 person-years for females) (Figure 2 and eTable 6 in Supplement 1). Males had a higher all-cause mortality across PIR quartiles compared with females with an adjusted MF HR of 1.57 (95% CI, 1.42-1.73) in the lowest income category or first quartile, 1.74 (95% CI, 1.51-2.00) in the second quartile, 1.78 (95% CI, 1.51-2.11) in the third quartile, and 1.35 (95% CI, 1.10-1.66) in the fourth quartile (*P* = .08 for interaction) (Figure 2 and eTable 7 in Supplement 1). The MF HR differed significantly by PIR for mortality due to cerebrovascular diseases (first quartile, 2.25 [95% CI, 1.31-3.86]; second quartile, 1.85 [95% CI, 1.02-3.37]; third quartile, 1.85 [95% CI, 0.92-3.72]; fourth quartile, 0.59 [95% CI, 0.25-1.38]; *P* = .03 for interaction) and for accidents (first quartile, 0.93 [95% CI, 0.57-1.52]; second quartile, 2.37 [95% CI, 1.25-4.50]; third quartile, 2.40 [95% CI, 1.03-5.58]; fourth quartile, 0.57 [95% CI, 0.16-1.99]; *P* = .02 for interaction). Males also had a consistently higher mortality risk from malignant neoplasms and heart disease in all PIR categories. The test for interaction found no difference for other MF HRs across PIR quartiles for specific causes of mortality.

### Self-Rated Health

All-cause mortality decreased with increasing self-rated health status. Those who rated their health as excellent included 686.9 (95% CI, 608.6-765.2) per 100 000 person-years for males and 510.1 (95% CI, 421.7-598.6) per 100 000 person-years for females; as very good, 810.5 (95% CI, 737.1-884.0) per 100 000 person-years for males and 736.4 (95% CI, 671.5- 801.4) per 100 000 person-years for females; as good, 1312.4 (95% CI, 1225.7-1399.2) per 100 000 person-years for males and 1124.5 (95% CI, 1033.5-1215.5) per 100 000 person-years for females; and as fair or poor, 2999.4 (95% CI, 2802.2-3196.7) per 100 000 person-years for males and 2486.7 (95% CI, 2306.6-2666.8) per 100 000 person-years for females (eFigure 1 and eTable 8 in Supplement 1). The 3 leading causes of mortality among males and females who perceived themselves to be in excellent or very good health were malignant neoplasms (220.8 [95% CI, 174.3-267.3] per 100 000 person-years for males and 153.5 [95% CI, 111.0-196.1] per 100 000 person-years for females), diseases of the heart (148.8 [95% CI, 109.7-187.8] per 100 000 person-years for males and 105.1 [95% CI, 69.9-140.2] per 100 000 person-years for females), and accidents for males (43.4 [95% CI, 18.3-68.5] per 100 000 person-years for males) and Alzheimer disease for females (34.6 [95% CI, 17.4-51.8] per 100 000 person-years for females). The top 3 causes of mortality among individuals who perceived their health to be fair or poor were diseases of the heart (832.2 [95% CI, 716.2-948.2] per 100 000 person-years for males and 669.5 [95% CI, 587.2-751.9] per 100 000 person-years for females), malignant neoplasms (644.6 [95% CI, 562.2-727.1] per 100 000 person-years for males and 416.4 [95% CI, 356.1-476.7] per 100 000 person-years for females), and chronic lower respiratory diseases (254.3 [95% CI, 194.5-314.0] per 100 000 person-years for males and 218.8 [95% CI, 170.9-266.7] per 100 000 person-years for females). When examining the MF HR for all-cause mortality across self-rated health categories, there was a higher risk of mortality among males compared with females regardless of self-perceived health status (eFigure 1 and eTable 9 in Supplement 1). Males had a consistently higher mortality risk for malignant neoplasms and heart disease, across self-rated health categories. The test for interaction found no difference in the MF HR across self-rated health category for specific mortality causes.

### Educational Attainment

All-cause mortality decreased with higher educational attainment for both males and females, with all-cause mortality rate of 1651.9 (95% CI, 1556.1-1747.7) per 100 000 person-years for males and 1636.0 (95% CI, 1541.7-1730.2) per 100 000 person-years for females among those with high school education or less, 1069.0 (95% CI, 977.7-1160.4) per 100 000 person-years for males and 918.6 (95% CI, 839.9-997.3) per 100 000 person-years for females among those with some college or an associate’s degree, and 861.5 (95% CI, 776.3-946.8) per 100 000 person-years for males and 521.0 (95% CI, 454.1-587.9) per 100 000 person-years for females among those with a college education or higher (eFigure 2 and eTable 10 in Supplement 1). The top 3 causes of mortality, among those in the lowest educational category were diseases of the heart (mortality rates, 431.0 [95% CI, 389.9-472.1] per 100 000 person-years for males and 404.8 [95% CI, 363.5-446.1] per 100 000 person-years for females), malignant neoplasms (mortality rates, 417.6 [95% CI, 369.7-465.5] per 100 000 person-years for males and 300.0 [95% CI, 262.2-337.9] per 100 000 person-years for females), and chronic lower respiratory diseases (mortality rates, 99.3 [95% CI, 80.5-118.1] per 100 000 person-years for males and 136.6 [95% CI, 111.0-162.2] per 100 000 person-years for females). Males had a higher all-cause mortality across all educational levels compared with females with an MF HR of 1.70 (95% CI, 1.56-1.86) for high school education or less, 1.53 (95% CI, 1.32-1.77) for some college or an associate’s degree, and 1.59 (95% CI, 1.30-1.94) for college graduates (*P* = .15 for interaction) (eFigure 2 and eTable 11 in Supplement 1). Males had a consistently higher mortality risk for diseases of the heart. The test for interaction found no difference in the MF HR across educational attainment for any specific causes of mortality.

## Discussion

In this cohort study of adults participating in NHANES, accounting for a wide range of risk factors, we found males had a 63% greater risk of all-cause mortality than females. Once sociodemographic characteristics, behavioral factors, and chronic conditions are accounted for, the results suggest that there are likely intrinsic biological sex differences (eg, sex hormones, chromosomes complement) that may be associated with increased mortality among males. Males had increased risks of mortality due to diseases of the heart, malignant neoplasms, cerebrovascular diseases, and diabetes compared with females. We found no significant differences in the sex gap in mortality across self-rated health or educational level. However, for diseases of the heart, the risk of mortality for males compared with females differed across race and ethnicity. Among White and Black individuals, males were 1.6 to 2.1 times more likely to die from diseases of the heart compared with their female counterparts, whereas there was no sex difference observed for Hispanic individuals or persons of other race or ethnicity. Differences in the MF HR were seen across PIR quartiles for cerebrovascular disease, where those in the lowest quartiles had the largest sex difference in mortality, and for accidents, where the second and third quartiles had the largest sex difference in mortality.

Traditionally, the sex gap in mortality has been attributed differences in sociodemographic characteristics, as well as lifestyle and behavioral factors. Women are more likely than men to engage in health-seeking behaviors such as routine health care visits, whereas men are more likely to engage in risky behaviors such as excessive alcohol consumption, risk taking, and high tobacco consumption.^19^ However, the past decades have seen a convergence in unhealthy behaviors between the sexes.^1^ For instance, in 1965, 51% of men and 34% of women were current cigarette smokers compared with 2023, when 13% of men and 9% of women reported current smoking.^20^ Similarly, between 2002 and 2012, the sex gap in alcohol use narrowed on all measures, including current drinking, number of drinking days per month, alcohol abuse, and driving under the influence of alcohol.^21^ Therefore, it is important for reports of sex differences in mortality rates to adjust for these risk factors directly rather than make conjecture about the factors driving the sex differences in mortality.

Other analyses of sex differences found comparable results to ours. Rogers et al^1^ conducted an analysis using NHANES III (1988-1994) and found that males had a 62% increased mortality risk compared with females after accounting for demographic characteristics, socioeconomic status, social relations, health behaviors, and chronic conditions.^1^ The authors concluded that as the health-seeking behaviors of males increased, the sex gap in mortality would decrease. However, despite a narrowing of the sex gap in harmful and healthful behaviors during the past 20 years,^20,21^ we found the sex gap in mortality unchanged. Using the 2005-2018 NHANES cycles, Lv et al^22^ investigated factors contributing to sex disparities in cardiovascular- and cerebrovascular-related mortality in the general population and in those with CVD. They found that males had a higher risk of both all-cause mortality and cardiovascular and cerebrovascular mortality in both populations after accounting for age, race and ethnicity, socioeconomic status, lifestyle and behavioral factors, comorbid conditions, and laboratory values. The fact that these disparities persisted after adjustment for all these factors led the authors to conclude that the sex gap may be due to biology or genetics. Results from a historical cohort of persons born between 1900 and 1935 found that smoking behavior accounted for only 30% of male excess mortality at ages 50 to 70 years, largely associated with CVD, suggesting that males have increased susceptibility above and beyond smoking.^23^ Finally, Wu et al^9^ found that males had a 60% higher risk of all-cause mortality compared with females after adjusting for age, socioeconomic, lifestyle, and health factors. However, this sex gap varied markedly by country with no sex difference seen in mortality in Mexico but a more than 2-fold increase in male mortality compared with female mortality in Japan.^9^ These geographical variations persisted even after adjusting for smoking and CVD.^9^

The persistence of a sex gap in mortality after adjusting for numerous risk factors related to environmental, social, and economic indicators of health suggests they may be intrinsic biological differences underlying the sex differences in in mortality.^1,9,22,24^ Biological risk factors such as biological aging, hormone levels, epigenetic changes, and immune responses can present differently in males and females, which may result in variation in life expectancy and mortality rates between sexes. Female adults tend to have longer telomere lengths, suggesting slower biological aging among females compared with males.^25,26^ Another study using age-related biomarkers across several domains, including cardiovascular, metabolic, and organ functioning, also found that females had significantly lower biological ages than males in each age-group comparison.^27^ Further research continues to observe sex differences in life expectancy, with females tending to have a greater life expectancy compared with males.^4,26,28,29^ After puberty, circulating testosterone and estrogen levels differ significantly between sexes.^30^ While testosterone can have a protective effect on cardiovascular health, having suboptimal testosterone levels is also associated with increases in cardiovascular events and mortality.^11,31,32,33^ Similarly, estrogen has a protective effect on cardiovascular health, and a drop in estrogen levels, especially in the postmenopause years, can increase the risk of CVD among women.^34,35^ In females, 1 of 2 X chromosomes is inactivated through the process of gene silencing. However, some X-linked genes escape inactivation, leading to overexpression of genes related to immune response in females, resulting in potentially differential susceptibility to disease between the sexes.^36,37^

### Strengths and Limitations

Our study has several important strengths, including our ability to link NHANES respondent data to the NDI, which is the gold standard of death ascertainment. The NHANES sample population is a representative sample of the US general population by design. Additionally, we were able to account for several socioeconomic, behavioral, lifestyle, and medical-related covariates in our analysis.

This study also has several limitations. Although the NDI has complete coverage of all deaths that occur in the US, a bias may occur if individuals died outside of the US; this may be why we did not see significant sex differences in mortality for individuals of other race or ethnicity (consisting largely of Asian American individuals), and to some extent Hispanic individuals, as these individuals may have been more likely to die outside the US. However, the estimates for White and Black individuals are unlikely to be subject to this same bias. Because these data were collected cross-sectionally, it is unknown whether an individual’s lifestyle at the time of interview remained constant over the time leading up to their death or end of follow up. Many of the covariates were collected from self-report (eg, daily alcohol consumption, cigarette smoking, physical activity, and sedentary behavior), resulting in underreporting or overreporting of these activities due to social desirability bias. There is also a possibility of overadjustment, with some covariates possibly being mediators instead of confounders (eg, marital status, income, chronic conditions, health insurance coverage).^22^ Finally, while NHANES is a population-based study, the response rate for participants who underwent physical examination was below 80% for 8 of 9 survey cycles.^38^ Nonetheless, there was no substantial difference in the response rates between sexes across the NHANES cycle.^38^

## Conclusions

In this cohort study, after accounting for multiple covariates that included risk factors and confounders, the gap in mortality between males and females remained for most causes of mortality. These findings suggest that biological factors (ie, sex hormone levels, epigenetic changes, and immune response) play an important role in mortality. More research needs to be conducted on the effects of sex-linked biological factors on mortality.

## Notes

Supplement 1. **eTable 1.** Underlying Cause of Mortality and Corresponding *ICD-10* Codes **eTable 2.** Harmonized National Health and Nutrition Examination Survey (NHANES) and National Death Index (NDI) Variables With Categorization (1999-2019) **eTable 3.** Cause-Specific Mortality Risk Factors and Their Corresponding Cox Models **eTable 4.** Weighted Male and Female Mortality Rates and 95% CIs per 100 000 by Cause of Mortality Within Each Racial and Ethnic Group **eTable 5.** Adjusted Male-to-Female Hazard Ratios for All-Cause and Top 9 Causes of Mortality by Race and Ethnicity **eTable 6.** Weighted Male and Female Mortality Rates and 95% CIs per 100 000 by Cause of Mortality Within Each Income-to-Poverty Ratio Category **eTable 7.** Adjusted Male-to-Female Hazard Ratios for All-Cause and Top 9 Causes of Mortality by Income-to-Poverty Ratio Quartiles **eFigure 1**. Weighted Male and Female Mortality Rates and Adjusted Male-to-Female Hazard Ratios for All-Cause and the Top 9 Causes of Mortality by Self-Rated Health, Ordered by Highest to Lowest Male Mortality Rates **eTable 8.** Weighted Male and Female Mortality Rates and 95% CIs per 100 000 by Cause of Mortality Within Self-Rated Health Category **eTable 9.** Adjusted Male-to-Female Hazard Ratios for All-Cause and the Top 9 Causes of Mortality by Self-Rated Health **eFigure 2**. Weighted Male and Female Mortality Rates and Adjusted Male-to-Female Hazard Ratios for All-Cause and the Top 9 Causes of Mortality by Highest Attained Education, Ordered by Highest to Lowest Common Male Mortality Rates **eTable 10.** Weighted Male and Female Mortality Rates and 95% CIs per 100 000 by Cause of Mortality Within Educational Category **eTable 11.** Adjusted Male-to-Female Hazard Ratios for All-Cause and Top 9 Causes of Mortality by Highest Educational Attainment

Supplement 2.
Data Sharing Statement
