# Supplementary material for: Sex and All-Cause Mortality in the US, 1999 to 2019
Source: JAMA Netw Open. 2026 Jan 30;9(1):e2556299. doi: 10.1001/jamanetworkopen.2025.56299 (PMC12859720; doi:10.1001/jamanetworkopen.2025.56299)
Supplement: Supplement 1. — eTable 1. Underlying Cause of Mortality and Corresponding ICD-10 Codes eTable 2. Harmonized National Health and Nutrition Examination Survey (NHANES) and National Death Index (NDI) Variables With Categorization (1999-2019) eTable 3. Cause-Specific Mortality Risk Factors and Their Corresponding Cox Models eTable 4. Weighted Male and Female Mortality Rates and 95% CIs per 100 000 by Cause of Mortality Within Each Racial and Ethnic Group eTable 5. Adjusted Male-to-Female Hazard Ratios for All-Cause and Top 9 Causes of Mortality by Race and Ethnicity eTable 6. Weighted Male and Female Mortality Rates and 95% CIs per 100 000 by Cause of Mortality Within Each Income-to-Poverty Ratio Category eTable 7. Adjusted Male-to-Female Hazard Ratios for All-Cause and Top 9 Causes of Mortality by Income-to-Poverty Ratio Quartiles eFigure 1. Weighted Male and Female Mortality Rates and Adjusted Male-to-Female Hazard Ratios for All-Cause and the Top 9 Causes of Mortality by Self-Rated Health, Ordered by Highest to Lowest Male Mortality Rates eTable 8. Weighted Male and Female Mortality Rates and 95% CIs per 100 000 by Cause of Mortality Within Self-Rated Health Category eTable 9. Adjusted Male-to-Female Hazard Ratios for All-Cause and the Top 9 Causes of Mortality by Self-Rated Health eFigure 2. Weighted Male and Female Mortality Rates and Adjusted Male-to-Female Hazard Ratios for All-Cause and the Top 9 Causes of Mortality by Highest Attained Education, Ordered by Highest to Lowest Common Male Mortality Rates eTable 10. Weighted Male and Female Mortality Rates and 95% CIs per 100 000 by Cause of Mortality Within Educational Category eTable 11. Adjusted Male-to-Female Hazard Ratios for All-Cause and Top 9 Causes of Mortality by Highest Educational Attainment [file jamanetwopen-e2556299-s001.pdf]

Supplementary Online Content

Francis J, Graubard BI, Katki H, Jackson SS. Sex and all-cause mortality in the US, 1999 to 2019. *JAMA Netw Open.* 2026;9(1):e2556299. doi:10.1001/jamanetworkopen.2025.56299

eTable 1. Underlying Cause of Mortality and Corresponding *ICD-10* Codes

eTable 2. Harmonized National Health and Nutrition Examination Survey (NHANES) and National Death Index (NDI) Variables With Categorization (1999-2019)

eTable 3. Cause-Specific Mortality Risk Factors and Their Corresponding Cox Models

eTable 4. Weighted Male and Female Mortality Rates and 95% CIs per 100 000 by Cause of Mortality Within Each Racial and Ethnic Group

eTable 5. Adjusted Male-to-Female Hazard Ratios for All-Cause and Top 9 Causes of Mortality by Race and Ethnicity

eTable 6. Weighted Male and Female Mortality Rates and 95% CIs per 100 000 by Cause of Mortality Within Each Income-to-Poverty Ratio Category

eTable 7. Adjusted Male-to-Female Hazard Ratios for All-Cause and Top 9 Causes of Mortality by Income-to-Poverty Ratio Quartiles

eFigure 1. Weighted Male and Female Mortality Rates and Adjusted Male-to-Female Hazard Ratios for All-Cause and the Top 9 Causes of Mortality by Self-Rated Health, Ordered by Highest to Lowest Male Mortality Rates

eTable 8. Weighted Male and Female Mortality Rates and 95% CIs per 100 000 by Cause of Mortality Within Self-Rated Health Category

eTable 9. Adjusted Male-to-Female Hazard Ratios for All-Cause and the Top 9 Causes of Mortality by Self-Rated Health

eFigure 2. Weighted Male and Female Mortality Rates and Adjusted Male-to-Female Hazard Ratios for All-Cause and the Top 9 Causes of Mortality by Highest Attained Education, Ordered by Highest to Lowest Common Male Mortality Rates

eTable 10. Weighted Male and Female Mortality Rates and 95% CIs per 100 000 by Cause of Mortality Within Educational Category

eTable 11. Adjusted Male-to-Female Hazard Ratios for All-Cause and Top 9 Causes of Mortality by Highest Educational Attainment

This supplementary material has been provided by the authors to give readers additional information about their work.

© 2026 Francis J et al. *JAMA Network Open*.

eTable 1. Underlying Cause of Mortality and Corresponding *ICD-10* Codes

**Cause of Mortality**	**ICD-10 Codes**
Malignant neoplasms	C00-C97
Diseases of heart	I00-I09, I11, I13, I20-I51
Chronic lower respiratory diseases	J40-J47
Cerebrovascular diseases	I60-I69
Accidents (unintentional injuries)	V01-X59, Y85-Y86
Diabetes mellitus	E10-E14
Alzheimer’s disease	G30
Nephritis, nephrotic syndrome and nephrosis	N00-N07, N17-N19, N25-N27
Influenza and pneumonia	J09-J18

© 2026 Francis J et al. *JAMA Network Open*.

**eTable 2.** Harmonized National Health and Nutrition Examination Survey (NHANES) and National Death Index (NDI) Variables With Categorization (1999-2019)				
**Concept**	**NHANES Variable Name**	**NHANES Question/Description**	**Francis *et al*. Variable Coding**	**Survey Years**
**DEMOGRAPHIC CHARACTERISTICS**				
**Sex**	RIAGENDR - Gender	Gender of the sample person<br><br>1 = Male<br>2 = Female<br>. = Missing	1 = Male<br>2 = Female	1999-2016
**Age in Years**	RIDAGEYR - Age in years at screening	Age in years of the participant at the time of screening. Individuals 85 and over are top coded at 85 years of age.<br><br>0 to 84 Range of Values<br>85 = >= 85 years of age<br>. = Missing	0 = 20-29 years<br>1 = 30-39 years<br>2 = 40-49 years<br>3 = 50-59 years<br>4 = 60-69 years<br>5 = 70-79 years<br>6 = 80+ years<br>9 = Under 20;	1999-2016
**Race/Ethnicity**	RIDRETH1 - Race/Ethnicity - Recode	Recode of reported race and ethnicity information.<br><br>1 = Mexican American<br>2 = Other Hispanic<br>3 = Non-Hispanic White<br>4 = Non-Hispanic Black<br>5 = Other Race (Including Multi-Racial)<br>. = Missing	1 = Non-Hispanic White<br>2 = Non-Hispanic Black<br>3 = Hispanic<br>4 = Other/Multi-Racial<br>. = Missing;	1999-2016
**Education Level**	DMDEDUC2 - Education Level - Adults 20+	What is the highest grade or level of school {you have/SP has} completed or the highest degree {you have/s/he has} received?	1 = High School or Less<br>2 = Some College or AA degree<br>3 = College Graduate or above<br>4= Missing	1999-2016

© 2026 Francis J et al. *JAMA Network Open*.

		1 = Less Than 9th Grade<br>2 = 9-11th Grade (Includes 12th grade with no diploma)<br>3 = High School Grad/GED or Equivalent<br>4 = Some College or AA degree<br>5 = College Graduate or above<br>7 = Refused<br>9 = Don't Know<br>. = Missing		
**Income to Poverty Ratio**	INDFMPIR - Family PIR	Poverty income ratio (PIR) - a ratio of family income to poverty threshold<br><br>0 to 4.99 Range of Values<br>5 = PIR value greater than or equal to 5.00<br>. = Missing	1 = 1st Quartile<br>2 = 2nd Quartile<br>3 = 3rd Quartile<br>4 = 4th Quartile<br>. = Missing	1999-2016
**Country of Birth**	DMDBORN - Country of Birth - Recode	In what country {were you/was SP} born?<br><br>1 = Born in 50 US States or Washington, DC<br>2 = Born in Mexico<br>3 = Born Elsewhere<br>7 = Refused<br>9 = Don't Know<br>. =Missing	1 = Born in the US<br>2 = Born elsewhere<br>. = Missing;	1999-2016
**Marital Status**	DMDMARTL - Marital Status	Marital Status	1 = Married/Living with Partner	1999-2016

© 2026 Francis J et al. *JAMA Network Open*.

		1 = Married<br>2 = Widowed<br>3 = Divorced<br>4 = Separated<br>5 = Never married<br>6 = Living with partner<br>77 = Refused<br>99 = Don't know<br>. = Missing	2 = Widowed/Separated/Divorced<br>3 = Never Married<br>. = Missing;	
**Occupation**	OCD240 - Occupation group code: current job	(SP Interview Version) What kind of work {were you/was SP} doing? (For example: farming, mail clerk, computer specialist.) (Family Interview Version) What kind of work {were/was} {you/NON-SP HEAD/NON-SP SPOUSE} doing? (For example: farming, mail clerk, computer specialist.)<br><br>1 to 98 Range of Values<br>. = Missing	1 = Non-hazardous, low physical activity<br>2 = Non-hazardous high physical activity<br>3 = Hazardous, high physical activity<br>4 = missing or unable to identify occupational exposure	1999-2004
	OCD241 – Occupation group code: current job	OCD241 What kind of work {were you/was SP} doing? (For example: farming, mail clerk, computer specialist.)<br><br>1 to 99 Range of Values<br>. = Missing		2005-2014
	OCQ260 – Description of job/work situation	Looking at the card, which of these best describes this job or work situation?		1999-2016

© 2026 Francis J et al. *JAMA Network Open*.

		1 = An employee of a private company, business, or individual for wages, salary, or commission.<br>2 = A federal government employee<br>3 = A state government employee<br>4 = A local government employee<br>5 = Self-employed in own business, professional practice or farm.<br>6 = Working without pay in family business or farm<br>77 = Refused<br>99 = Don't know<br>. = Missing		
	OCD390G - Kind of work you have done the longest	Thinking of all the paid jobs or businesses {you/SP} ever had, what kind of work {were you/was s/he} doing the longest? (For example, electrical engineer, stock clerk, typist, farmer.)<br><br>1 = Enter occupation<br>2 = Same as current occupation<br>3 = Armed forces<br>4 = Never worked<br>7 = Refused<br>9 = Don't know<br>. = Missing		2001-2016

© 2026 Francis J et al. *JAMA Network Open*.

HEALTH CHARACTERISTICS				
**Body Mass Index (kg/m²)**	BMXBMI-Body Mass Index (kg/m²)	Body Mass Index (kg/m²)<br><br>Range of Values	1 = Normal<br>2 = Underweight<br>3 = Overweight<br>4 = Obese<br>. = Missing;	1999-2016
**General Health**	HUQ010 - General health condition	{First/Next} I have some general questions about {your/SP's} health. Would you say {your/SP's} health in general is . . .<br><br>1 = Excellent<br>2 = Very Good<br>3 = Good<br>4 = Fair<br>5 = Poor<br>. = Missing	1 = Excellent<br>2 = Very Good<br>3 = Good<br>4 = Fair/Poor<br>. = Missing;	1999-2016
**Health Insurance Coverage**	HIQ011 - Covered by health insurance	The (first/next) questions are about health insurance. {Are you/Is SP} covered by health insurance or some other kind of health care plan? [Include health insurance obtained through employment or purchased directly as well as government programs like Medicare and Medicaid that provide medical care or help pay medical bills.]<br><br>1 = Yes<br>2 = No<br>7 = Refused	1 = Insured<br>2 = Uninsured<br>. = Missing;	2005-2016

© 2026 Francis J et al. *JAMA Network Open*.

		9 = Don't know<br>. = Missing		
	HID010 - Covered by health insurance	The (first/next) questions are about health insurance. {Are you/Is SP} covered by health insurance or some other kind of health care plan? [Include health insurance obtained through employment or purchased directly as well as government programs like Medicare and Medicaid that provide medical care or help pay medical bills.]<br><br>1 = Yes<br>2 = No<br>7 = Refused<br>9 = Don't know<br>. = Missing		1999-2004
**Routine place to go for healthcare**	HUQ030 - Routine place to go for healthcare	Is there a place that {you/SP} usually {go/goes} when {you are/he/she is} sick or {you/s/he} need{s} advice about {your/his/her} health?<br><br>1 = Yes<br>2 = There is no place<br>3 = There is more than one place<br>7 = Refused<br>9 = Don't know<br>. = Missing	1 = Yes<br>2 = No<br>. = Missing	1999-2016

© 2026 Francis J et al. *JAMA Network Open*.

**History of Heart Disease**	MCQ160C - Ever told you had coronary heart disease	Has a doctor or other health professional ever told {you/SP} that {you/s/he} . . .had coronary (kor-o-nare-ee) heart disease?<br><br>1 = Yes<br>2 = No<br>7 = Refused<br>9 = Don't know<br>. = Missing	1 = Yes<br>0 = No<br>. = Missing	1999-2016
**History of Diabetes**	DIQ010 - Doctor told you have diabetes	The next questions are about specific medical conditions. {Other than during pregnancy, {have you/has SP}/{Have you/Has SP}} ever been told by a doctor or health professional that {you have/{he/she/SP} has} diabetes or sugar diabetes?<br><br>1 = Yes<br>2 = No<br>3 = Borderline<br>7 = Refused<br>9 = Don't know<br>. = Missing	1 = Yes<br>0 = No/borderline<br>. = Missing	1999-2016
**History of Hypertension**	BPQ020 - Ever told you had high blood pressure	{Have you/Has SP} ever been told by a doctor or other health professional that {you/s/he} had hypertension, also called high blood pressure?	1 = Yes<br>0 = No<br>. = Missing	1999-2016

© 2026 Francis J et al. *JAMA Network Open*.

		1 = Yes<br>2 = No<br>7 = Refused<br>9 = Don't know<br>. = Missing		
**History of Cancer**	MCQ220 - Ever told you had cancer or malignancy	{Have you/Has SP} ever been told by a doctor or other health professional that {you/s/he} had cancer or a malignancy of any kind?<br><br>1 = Yes<br>2 = No<br>7 = Refused<br>9 = Don’t Know<br>. = Missing	1 = Yes<br>2 = No<br>. = Missing	1999-2016
**LIFESTYLE CHARACTERISTICS**				
**Number of Alcoholic Drinks/Day**	ALQ130 - Avg # alcoholic drinks/day - past 12 mos	In the past 12 months, on those days that {you/SP} drank alcoholic beverages, on the average, how many drinks did {you/he/she} have?<br><br>IF LESS THAN 1 DRINK, ENTER '1'. IF 95 DRINKS OR MORE, ENTER '95'. ENTER # OF DRINKS.	1 = One or less (Little to none)<br>2 = >1 drinks to ≤ 2 (Moderate)<br>3 = >2 drinks to ≤ 12 (Heavy)<br>. = Missing/ More than 12 Drinks	1999-2016
**Smoking Intensity**	SMQ020 - Smoked at least 100 cigarettes in life	These next questions are about cigarette smoking and other tobacco use. {Have you/Has SP} smoked at least 100 cigarettes in {your/his/her} entire life?	1 = Non-Smoker<br>2 = Past Smoker, <10 cigarettes<br>3 = Past Smoker, ≥10 cigarettes<br>4 = Current Smoker, <10 cigarettes	1999-2016

© 2026 Francis J et al. *JAMA Network Open*.

		1=Yes<br>2=No<br>7=Refused<br>9=Don’t know<br>.=Missing	5 = Current Smoker, ≥10 cigarettes<br>. = Missing	
	SMQ040 - Do you now smoke cigarettes	{Do you/Does SP} now smoke cigarettes…<br><br>1=Every day<br>2=Somedays<br>3=Not at all<br>7=Refused<br>9=Don’t know<br>.=Missing		1999-2016
	SMD090 - # cigarettes smoked per day now	During the past 30 days, on the days that {you/SP} smoked, about how many cigarettes did {you/s/he} smoke per day?<br><br>Range of Values 1 to 90<br>777=Refused<br>999=Don't know<br>.=Missing		1999-2002
	SMD650 - Avg # cigarettes/day during past 30 days	During the past 30 days, on the days that {you/SP} smoked, about how many cigarettes did {you/s/he} smoke per day?<br><br>Range of Values<br>95=95 cigarettes or more<br>777=Refused<br>999=Don't know		2003-2016

© 2026 Francis J et al. *JAMA Network Open*.

		.=Missing		
**Physical Activity**	PAD320 - Moderate activity over past 30 days	[Over the past 30 days], did {you/SP} do moderate activities for at least 10 minutes that cause only light sweating or a slight to moderate increase in breathing or heart rate? Some examples are brisk walking, bicycling for pleasure, golf, and dancing.<br><br>1=Yes<br>2=No<br>3=Unable to exercise<br>7=Refused<br>9=Don't know<br>.=Missing	1 = Yes<br>0 = No/Unable to Exercise<br>. = Missing	1999-2006
	PAQ665 - Moderate recreational activities	{Do you/Does SP} do any moderate-intensity sports, fitness, or recreational activities that cause a small increase in breathing or heart rate such as brisk walking, bicycling, swimming, or golf for at least 10 minutes continuously?<br><br>1=Yes<br>2=No<br>7=Refused<br>9=Don't know<br>.=Missing		2007-2016

© 2026 Francis J et al. *JAMA Network Open*.

**Daily Hours of Sedentary Behavior**	PAD480 and PAQ- Daily hours of TV, video or computer use	Now I will ask about TV watching or computer use. Over the past 30 days, on a typical day how much time altogether did {you/SP} spend on a typical day sitting and watching TV or videos or using a computer outside of work? Would you say…<br><br>0=Less than 1 hour<br>1=1 hour<br>2=2 hours<br>3=3 hours<br>4=4 hours<br>5=5 hours or more<br>6=None<br>77=Refused<br>99=Don't know<br>.=Missing	1 = None to <1 hour<br>2 = 2 hours or less<br>3 = 3 to 4 hours<br>4 = 5 hours or more<br>. = Missing	1999-2002
	PAD590 - Hours watch TV or videos past 30 days	Over the past 30 days, on average how many hours per day did {SP} sit and watch TV or videos? Would you say...<br><br>0=Less than 1 hour<br>1=1 hour<br>2=2 hours<br>3=3 hours<br>4=4 hours<br>5=5 hours or more<br>6=None<br>77=Refused<br>99=Don't know		2003-2010

© 2026 Francis J et al. *JAMA Network Open*.

		.=Missing		
	PAD600 - Hours use computer past 30 days	Over the past 30 days, on average how many hours per day did {SP} use a computer or play computer games outside of school? Would you say...<br><br>0=Less than 1 hour<br>1=1 hour<br>2=2 hours<br>3=3 hours<br>4=4 hours<br>5=5 hours or more<br>6=None<br>77=Refused<br>99=Don't know<br>.=Missing		2003-2010
	PAQ710 - Hours watch TV or videos past 30 days	Now I will ask you first about TV watching and then about computer use. Over the past 30 days, on average how many hours per day did {you/SP} sit and watch TV or videos? Would you say…<br><br>0=Less than 1 hour<br>1=1 hour<br>2=2 hours<br>3=3 hours<br>4=4 hours<br>5=5 hours or more<br>6=None		2011-2016

© 2026 Francis J et al. *JAMA Network Open*.

		8={You do/SP does} not watch TV or videos<br>77=Refused<br>99=Don't know<br>.=Missing		
	PAQ715 - Hours use computer past 30 days	Over the past 30 days, on average how many hours per day did {you/SP} use a computer or play computer games outside of work or school? Include PlayStation, Nintendo DS, or other portable video games. Would you say…<br><br>0=Less than 1 hour<br>1=1 hour<br>2=2 hours<br>3=3 hours<br>4=4 hours<br>5=5 hours or more<br>6=None<br>8={You do/SP does} not watch TV or videos<br>77=Refused<br>99=Don't know<br>.=Missing		2011-2016
	PAD680 - Minutes sedentary activity	The following question is about sitting at work, at home, getting to and from places, or with friends, including time spent sitting at a desk, traveling in a car or bus, reading, playing cards,		2007-2016

© 2026 Francis J et al. *JAMA Network Open*.

		watching television, or using a computer. Do not include time spent sleeping. How much time {do you/does SP} usually spend sitting on a typical day?<br><br>0 to 1380=Range of Values<br>7777=Refused<br>9999=Don't know<br>.=Missing		

© 2026 Francis J et al. *JAMA Network Open*.

eTable 3. Cause-Specific Mortality Risk Factors and Their Corresponding Cox Models

Covariate*	Model (Cause of Mortality)
**Sedentary Behavior**	Diseases of the heart<br>Chronic lower respiratory diseases<br>Accidents (unintentional injuries)<br>Cerebrovascular diseases<br>Diabetes mellitus<br>Influenza/pneumonia
**Physical Activity**	Diseases of the heart<br>Malignant neoplasms<br>Alzheimer's disease<br>Diabetes mellitus<br>Nephritis, nephrotic syndrome and nephrosis
**History of Cardiovascular disease**	Diseases of the heart<br>Cerebrovascular diseases<br>Influenza/pneumonia<br>Nephritis, nephrotic syndrome and nephrosis
**History of Hypertension**	Diseases of the heart<br>Cerebrovascular diseases<br>Diabetes mellitus<br>Influenza/pneumonia<br>Nephritis, nephrotic syndrome and nephrosis
**History of Diabetes**	Diabetes mellitus<br>Nephritis, nephrotic syndrome and nephrosis
**History of Cancer**	Malignant neoplasms<br>Diabetes mellitus

*All Cox Proportional Hazard models, regardless of cause of mortality, adjusted for age, race/ethnicity, education, family income-to-poverty ratio, health insurance coverage, marital status, routine place to go for healthcare, occupation, smoking intensity, alcohol, body mass index

© 2026 Francis J et al. *JAMA Network Open*.

**eTable 4.** Weighted Male and Female Mortality Rates and 95% CIs per 100 000 by Cause of Mortality Within Each Racial and Ethnic Group		
**Cause of Mortality**	**Male Mortality rate per 100,000 (95% CI)**	**Female Mortality rate per 100,000 (95% CI)**
**Non-Hispanic White**		
All-cause mortality	1,424.0 (1,348.2, 1,499.7)	1256.4 (1183.2, 1329.6)
Malignant neoplasms	375.8 (337.3, 414.4)	256.6 (227.6, 285.6)
Diseases of heart	366.1 (330.6, 401.7)	295.4 (259.1, 331.6)
Chronic lower respiratory diseases	81.5 (65.8, 97.2)	103.0 (86.0, 120.0)
Cerebrovascular diseases	67.0 (53.9, 80.2)	65.6 (52.0, 79.3)
Accidents (unintentional injuries)	61.7 (61.7, 61.7)	61.7 (34.0, 58.2)
Diabetes mellitus	44.7 (32.7, 56.7)	38.2 (27.7, 48.7)
Alzheimer's disease	36.6 (28.2, 45.1)	70.3 (58.6, 81.9)
Nephritis, nephrotic syndrome and nephrosis	23.5 (15.2, 31.8)	26.0 (17.9, 34.1)
Influenza and pneumonia	21.1 (13.8, 28.3)	29.8 (19.4, 40.1)
**Non-Hispanic Black**		
All-cause mortality	1,350.3 (1,236.4, 1,464.1)	1,082.6 (999.9, 1,165.3)
Malignant neoplasms	364.9 (312.2, 417.6)	218.3 (183.8, 252.9)
Diseases of heart	346.7 (293.6, 399.8)	299.7 (252.0, 347.3)
Chronic lower respiratory diseases	49.9 (33.5, 66.3)	28.5 (16.7, 40.2)
Cerebrovascular diseases	60.5 (39.8, 81.1)	60.9 (44.5, 77.4)
Accidents (unintentional injuries)	47.3 (22.3, 72.4)	20.7 (8.7, 32.6)
Diabetes mellitus	45.4 (28.5, 62.3)	56.4 (36.4, 76.3)
Alzheimer's disease	21.0 (10.6, 31.4)	31.9 (20.6, 43.3)
Nephritis, nephrotic syndrome and nephrosis	39.5 (22.8, 56.3)	46.2 (27.8, 64.6)
Influenza and pneumonia	21.8 (10.8, 32.9)	15.3 (5.3, 25.2)
**Hispanic**		
All-cause mortality	635.4 (533.2, 737.6)	601.1 (459.3, 742.9)
Malignant neoplasms	145.6 (118.6, 172.6)	125.9 (94.7, 157.1)
Diseases of heart	133.8 (100.0, 167.6)	140.4 (100.7, 180.1)
Chronic lower respiratory diseases	23.5 (7.5, 39.6)	11.1 (4.2, 17.9)
Cerebrovascular diseases	34.0 (20.2, 47.9)	41.0 (20.6, 61.4)
Accidents (unintentional injuries)	32.1 (12.8. 51.5)	41.5 (4.5, 78.4)
Diabetes mellitus	44.8 (26.3, 63.2)	30.3 (14.9, 45.6)
Alzheimer's disease	13.5 (5.2, 21.8)	14.6 (5.5, 23.8)
Nephritis, nephrotic syndrome and nephrosis	21.8 (7.1, 36.4)	8.0 (1.8, 14.2)
Influenza and pneumonia	15.9 (5.1, 26.7)	10.6 (2.2, 19.0)
**Another Race**		
All-cause mortality	809.5 (643.0, 975.9)	720.3 (578.0, 862.7)
Malignant neoplasms	183.0 (98.0, 268.0)	127.3 (66.0, 188.6)
Diseases of heart	118.2 (59.6, 176.9)	186.5 (113.4, 259.6)
Chronic lower respiratory diseases	75.5 (29.1, 122.0)	35.3 (3.4, 67.1)
Cerebrovascular diseases	84.4 (31.4, 137.3)	25.6 (1.7, 49.5)
Accidents (unintentional injuries)	22.6 (-3.2, 48.3)	16.5 (-3.9, 36.9)

© 2026 Francis J et al. *JAMA Network Open*.

Diabetes mellitus	29.9 (2.3, 57.4)	37.1 (12.4, 61.7)
Alzheimer's disease	36.7 (4.1, 69.4)	16.1 (-0.4, 32.6)
Nephritis, nephrotic syndrome and nephrosis	8.9 (-4.1, 22.0)	10.6 (-3.0, 24.3)
Influenza and pneumonia	15.7 (7.7, 23.8)	17.5 (-7.6, 42.7)

© 2026 Francis J et al. *JAMA Network Open*.

eTable 5. Adjusted Male-to-Female Hazard Ratios for All-Cause and Top 9 Causes of Mortality by Race and Ethnicity

Cause of Mortality	Fully Adjusted MF HR (95% CI)				*P*-value[†]
	Non-Hispanic White	Non-Hispanic Black	Hispanic	Another Race*	
All-cause mortality[‡]	1.68 (1.54, 1.82)	1.46 (1.31, 1.64)	1.55 (1.28, 1.88)	1.21 (0.90, 1.63)	0.63
Malignant neoplasms[§]	1.69 (1.43, 2.00)	1.73 (1.33, 2.25)	1.68 (1.15, 2.45)	1.12 (0.51, 2.45)	0.98
Diseases of heart[‖]	2.11 (1.80, 2.48)	1.55 (1.21, 1.99)	1.47 (0.85, 2.54)	0.92 (0.36, 2.35)	0.02
Chronic lower respiratory diseases[¶]	0.90 (0.67, 1.21)	1.55 (0.87, 2.76)	1.58 (0.63, 3.92)	1.63 (0.31, 8.52)	0.38
Cerebrovascular diseases**	1.81 (1.22, 2.68)	1.60 (0.80, 3.21)	1.23 (0.57, 2.65)	6.07 (0.47, 78.62)	0.90
Accidents (unintentional injuries)[††]	1.50 (0.99, 2.26)	2.12 (0.68, 6.60)	1.28 (0.50, 3.31)	0.55 (0.07, 4.36)	0.40
Diabetes mellitus[‡‡]	1.85 (1.12, 3.07)	0.96 (0.47, 1.97)	5.02 (2.27, 11.13)	4.92 (0.21, 241.82)	0.18
Alzheimer's disease[§§]	1.05 (0.72, 1.53)	0.86 (0.34, 2.14)	2.12 (0.95, 4.73)	116.47 (7.63, 1915.49)	0.22
Nephritis, nephrotic syndrome and nephrosis[‖‖]	1.47 (0.80, 2.70)	1.18 (0.58, 2.38)	3.53 (1.38, 9.00)	2.61 (0.00, 14708.31)	0.42
Influenza and pneumonia**	1.50 (0.79, 2.87)	1.20 (0.42, 3.85)	1.82 (0.87, 3.85)	2.17 (0.18, 26.11)	0.51

Abbreviations: CI, confidence interval and MF HR, male-to-female hazard ratios

*Another race includes Asian American individuals, multiracial individuals and individuals who do not identify as Non-Hispanic White, Non-Hispanic Black or Hispanic

†*P*-value from Wald test assessing the statistical significance of the estimated interaction term for sex by race/ethnicity in the Cox proportional hazards model

‡age, age^2, race/ethnicity, education, family income to poverty ratio, health insurance coverage, marital status, routine place to go for healthcare, occupation, smoking intensity, alcohol, body mass index, sedentary behavior, physical activity, history of cardiovascular disease, history of hypertension, history of diabetes, history of cancer

§age, age^2, race/ethnicity, education, family income to poverty ratio, health insurance coverage, marital status, routine place to go for healthcare, occupation, smoking intensity, alcohol, body mass index, physical activity, history of cancer

‖age, age^2, race/ethnicity, education, family income to poverty ratio, health insurance coverage, marital status, routine place to go for healthcare, occupation, smoking intensity, alcohol, body mass index, sedentary behavior, physical activity, history of cardiovascular disease, history of hypertension

¶age, age^2, race/ethnicity, education, family income to poverty ratio, health insurance coverage, marital status, routine place to go for healthcare, occupation, smoking intensity, alcohol, body mass index, sedentary behavior

**age, age^2, race/ethnicity, education, family income to poverty ratio, health insurance coverage, marital status, routine place to go for healthcare, occupation, smoking intensity, alcohol, body mass index, sedentary behavior, history of cardiovascular disease, history of hypertension

††age, age^2, race/ethnicity, education, family income to poverty ratio, health insurance coverage, marital status, routine place to go for healthcare, occupation, smoking intensity, alcohol, body mass index, , sedentary behavior

‡‡age, age^2, race/ethnicity, education, family income to poverty ratio, health insurance coverage, marital status, routine place to go for healthcare, occupation, smoking intensity, alcohol, body mass index, sedentary behavior, physical activity, history of hypertension, history of diabetes, history of cancer

§§age, age^2, race/ethnicity, education, family income to poverty ratio, health insurance coverage, marital status, routine place to go for healthcare, occupation, smoking intensity, alcohol, body mass index, physical activity

‖‖age, age^2, race/ethnicity, education, family income to poverty ratio, health insurance coverage, marital status, routine place to go for healthcare, occupation, smoking intensity, alcohol, body mass index, physical activity, history of cardiovascular disease, history of hypertension, history of diabetes

© 2026 Francis J et al. *JAMA Network Open*.

**eTable 6.** Weighted Male and Female Mortality Rates and 95% CIs per 100 000 by Cause of Mortality Within Each Income-to-Poverty Ratio Category		
**Cause of Mortality**	**Male Mortality rates per 100,000 (95% CI)**	**Female Mortality rates per 100,000 (95% CI)**
**1st Quartile**		
All-cause mortality	1,590.6 (1,477.9, 1,703.4)	1,615.9 (1,502.1, 1,729.7)
Malignant neoplasms	358.4 (305.3, 411.5)	291.0 (247.8, 334.2)
Diseases of heart	410.8 (355.9, 465.8)	458.0 (396.2, 519.7)
Chronic lower respiratory diseases	92.8 (68.5, 117.2)	108.5 (79.9, 137.1)
Cerebrovascular diseases	82.3 (56.7, 107.9)	61.4 (44.0, 78.8)
Accidents (unintentional injuries)	50.9 (34.1, 67.7)	70.5 (42.1, 99.0)
Diabetes mellitus	58.7 (33.8, 78.6)	49.5 (31.4, 67.5)
Alzheimer's disease	31.1 (18.5, 43.7)	54.6 (37.5, 71.7)
Nephritis, nephrotic syndrome and nephrosis	31.2 (19.4, 43.1)	42.8 (27.0, 58.6)
Influenza and pneumonia	35.1 (24.1, 46.1)	30.7 (15.3, 46.1)
**2nd Quartile**		
All-cause mortality	1,700.3 (1,575.5, 1,825.1)	1,375.6 (1,271.9, 1,479.4)
Malignant neoplasms	403.1 (348.8, 457.4)	246.6 (203.2, 290.1)
Diseases of heart	439.6 (379.0.6, 500.2)	319.8 (267.5, 372.2)
Chronic lower respiratory diseases	91.3 (67.6, 114.9)	118.1 (89.6, 146.5)
Cerebrovascular diseases	84.8 (60.9, 108.7)	79.8 (53.9, 105.7)
Accidents (unintentional injuries)	74.5 (44.5, 104.4)	36.8 (19.1, 54.4)
Diabetes mellitus	58.4 (12.1, 82.4)	52.5 (32.4, 72.6)
Alzheimer's disease	36.1 (22.8, 49.4)	77.9 (55.6, 100.1)
Nephritis, nephrotic syndrome and nephrosis	36.7 (19.6, 53.8)	32.5 (14.6, 50.4)
Influenza and pneumonia	23.8 (13.2, 34.5)	33.1 (16.5, 49.6)
**3rd Quartile**		
All-cause mortality	1,089.3 (997.0, 1,181.6)	750.8 (662.9, 838.7)
Malignant neoplasms	300.4 (241.4, 359.3)	198.1 (155.0, 241.3)
Diseases of heart	279.2 (233.7, 324.7)	166.3 (126.9, 205.8)
Chronic lower respiratory diseases	58.8 (34.6, 83.1)	43.3 (23.8, 62.8)
Cerebrovascular diseases	57.2 (38.6, 75.9)	37.6 (21.7, 53.5)
Accidents (unintentional injuries)	72.2 (39.1, 105.3)	28.9 (9.1, 48.7)
Diabetes mellitus	32.8 (15.7, 49.9)	27.6 (9.7, 45.4)
Alzheimer's disease	36.6 (20.8, 52.4)	49.5 (31.6, 67.3)
Nephritis, nephrotic syndrome and nephrosis	16.8 (5.6, 28.0)	13.7 (6.0, 21.3)
Influenza and pneumonia	16.3 (4.1, 28.4)	16.7 (4.5, 28.8)
**4th Quartile**		
All-cause mortality	726.4 (646.8, 806.0)	555.2 (487.1, 623.4)
Malignant neoplasms	252.2 (196.3, 308.0)	164.2 (122.7, 205.7)
Diseases of heart	177.8 (136.9, 218.8)	84.7 (59.8, 109.6)
Chronic lower respiratory diseases	24.3 (12.9, 35.8)	31.0 (12.9, 49.2)
Cerebrovascular diseases	24.3 (11.9, 36.7)	40.4 (22.5, 58.3)
Accidents (unintentional injuries)	16.9 (5.0, 28.7)	28.5 (8.9, 48.1)

© 2026 Francis J et al. *JAMA Network Open*.

Diabetes mellitus	25.1 (8.1, 42.2)	17.3 (4.1, 30.6)
Alzheimer's disease	17.0 (8.3, 25.7)	31.6 (16.5, 46.8)
Nephritis, nephrotic syndrome and nephrosis	12.2 (4.9, 19.5)	9.1 (0.9, 17.3)
Influenza and pneumonia	6.0 (0.1, 11.8)	11.0 (2.2, 19.8)

© 2026 Francis J et al. *JAMA Network Open*.

**eTable 7.** Adjusted Male-to-Female Hazard Ratios for All-Cause and Top 9 Causes of Mortality by Income-to-Poverty Ratio Quartiles					
	**Fully Adjusted MF HR (95% CI)**				***P*-value***
**Cause of Mortality**	**1st Quartile (lowest income)**	**2nd Quartile**	**3rd Quartile**	**4th Quartile (highest income)**	
All-cause mortality†	1.57 (1.42, 1.73)	1.74 (1.51, 2.00)	1.78 (1.51, 2.11)	1.35 (1.10, 1.66)	0.08
Malignant neoplasms‡	1.62 (1.27, 2.08)	1.90 (1.44, 2.50)	1.64 (1.17, 2.30)	1.65 (1.14, 2.39)	0.24
Diseases of heart§	1.56 (1.27, 1.91)	2.03 (1.61, 2.57)	2.39 (1.75, 3.27)	2.24 (1.37, 3.64)	0.25
Chronic lower respiratory diseases‖	1.22 (0.83, 1.81)	0.75 (0.45, 1.27)	1.61 (0.74, 3.52)	0.37 (0.15, 0.92)	0.24
Cerebrovascular diseases¶	2.25 (1.31, 3.86)	1.85 (1.02, 3.37)	1.85 (0.92, 3.72)	0.59 (0.25, 1.38)	0.03
Accidents (unintentional injuries)**	0.93 (0.57, 1.52)	2.37 (1.25, 4.50)	2.40 (1.03, 5.58)	0.57 (0.16, 1.99)	0.02
Diabetes mellitus††	2.07 (1.26, 3.40)	1.99 (0.97, 4.07)	1.55 (0.45, 5.35)	1.44 (0.36, 5.77)	0.87
Alzheimer's disease‡‡	1.64 (0.92, 2.91)	1.34 (0.82, 2.21)	1.08 (0.58, 2.00)	0.56 (0.18, 1.73)	0.49
Nephritis, nephrotic syndrome and nephrosis§§	1.40 (0.73, 2.71)	1.25 (0.47, 3.31)	2.18 (0.78, 6.11)	2.09 (0.59, 7.43)	0.85
Influenza and pneumonia¶	1.43 (0.47, 4.30)	1.84 (0.76, 4.44)	1.42 (0.50, 4.02)	0.91 (0.34, 2.42)	0.82

Abbreviations: CI, confidence interval and MF HR, male-to-female hazard ratios

**P*-value from Wald test assessing the statistical significance of the estimated interaction term for sex by income-to-poverty ratio in the Cox proportional hazards model

†age, age^2, race/ethnicity, education, family income to poverty ratio, health insurance coverage, marital status, routine place to go for healthcare, occupation, smoking intensity, alcohol, body mass index, sedentary behavior, physical activity, history of cardiovascular disease, history of hypertension, history of diabetes, history of cancer

‡age, age^2, race/ethnicity, education, family income to poverty ratio, health insurance coverage, marital status, routine place to go for healthcare, occupation, smoking intensity, alcohol, body mass index, physical activity, history of cancer

§age, age^2, race/ethnicity, education, family income to poverty ratio, health insurance coverage, marital status, routine place to go for healthcare, occupation, smoking intensity, alcohol, body mass index, sedentary behavior, physical activity, history of cardiovascular disease, history of hypertension

‖age, age^2, race/ethnicity, education, family income to poverty ratio, health insurance coverage, marital status, routine place to go for healthcare, occupation, smoking intensity, alcohol, body mass index, sedentary behavior

¶age, age^2, race/ethnicity, education, family income to poverty ratio, health insurance coverage, marital status, routine place to go for healthcare, occupation, smoking intensity, alcohol, body mass index, sedentary behavior, history of cardiovascular disease, history of hypertension

**age, age^2, race/ethnicity, education, family income to poverty ratio, health insurance coverage, marital status, routine place to go for healthcare, occupation, smoking intensity, alcohol, body mass index, , sedentary behavior

††age, age^2, race/ethnicity, education, family income to poverty ratio, health insurance coverage, marital status, routine place to go for healthcare, occupation, smoking intensity, alcohol, body mass index, sedentary behavior, physical activity, history of hypertension, history of diabetes, history of cancer

‡‡age, age^2, race/ethnicity, education, family income to poverty ratio, health insurance coverage, marital status, routine place to go for healthcare, occupation, smoking intensity, alcohol, body mass index, physical activity

§§age, age^2, race/ethnicity, education, family income to poverty ratio, health insurance coverage, marital status, routine place to go for healthcare, occupation, smoking intensity, alcohol, body mass index, physical activity, history of cardiovascular disease, history of hypertension, history of diabetes

© 2026 Francis J et al. *JAMA Network Open*.

eFigure 2. Weighted Male and Female Mortality Rates and Adjusted Male-to-Female Hazard Ratios for All-Cause and the Top 9 Causes of Mortality by Self-Rated Health, Ordered by Highest to Lowest Male Mortality Rates
Key: Blue bars represent male mortality rates; red bars represent female mortality rates; boxes indicate male-to-female hazard ratios; whiskers indicate 95% confidence intervals; and dotted line represents the line of unity.

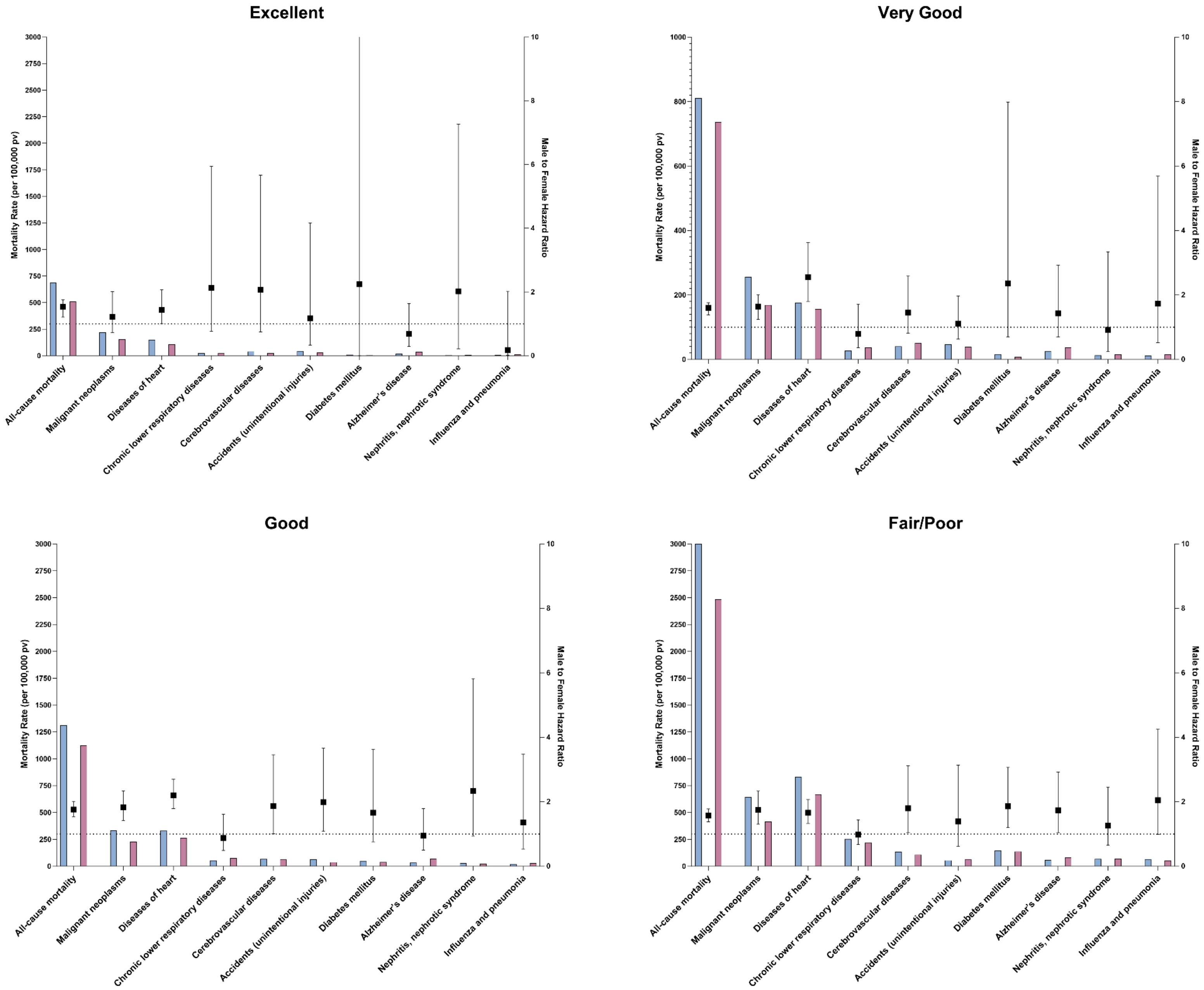

© 2026 Francis J et al. *JAMA Network Open*.

**eTable 8.** Weighted Male and Female Mortality Rates and 95% CIs per 100 000 by Cause of Mortality Within Self-Rated Health Category		
**Cause of Mortality**	**Male Mortality rates per 100,000 (95% CI)**	**Female Mortality rates per 100,000 (95% CI)**
**Excellent**		
All-cause mortality	686.9 (608.6, 765.2)	510.1 (421.7, 598.6)
Malignant neoplasms	220.8 (174.3, 267.3)	153.5 (111.0, 196.1)
Diseases of heart	148.8 (109.7, 187.8)	105.1 (69.9, 140.2)
Chronic lower respiratory diseases	25.5 (9.7, 41.2)	23.0 (9.0, 37.0)
Cerebrovascular diseases	39.8 (21.6, 58.0)	25.4 (10.5, 40.3)
Accidents (unintentional injuries)	43.4 (18.3, 68.5)	29.5 (6.1, 52.9)
Diabetes mellitus	9.4 (1.3, 17.6)	3.6 (-1.5, 8.8)
Alzheimer's disease	19.7 (7.8, 31.6)	34.6 (17.4, 51.8)
Nephritis, nephrotic syndrome and nephrosis	4.7 (0.1, 9.4)	6.1 (-0.5, 12.8)
Influenza and pneumonia	5.8 (0.9, 10.8)	11.8 (0.9, 22.7)
**Very Good**		
All-cause mortality	810.5 (737.1, 884.0)	736.4 (671.5, 801.4)
Malignant neoplasms	255.5 (207.3, 303.7)	168.8 (134.5, 203.1)
Diseases of heart	175.9 (142.6, 209.3)	156.8 (125.5, 188.1)
Chronic lower respiratory diseases	27.5 (15.2, 39.7)	36.7 (21.5, 51.9)
Cerebrovascular diseases	40.7 (23.5, 57.8)	50.5 (34.3, 66.8)
Accidents (unintentional injuries)	47.1 (29.5, 64.8)	39.8 (25.0, 54.7)
Diabetes mellitus	16.3 (6.2, 26.3)	8.0 (3.1, 12.9)
Alzheimer's disease	25.9 (16.9, 34.9)	36.7 (24.4, 49.1)
Nephritis, nephrotic syndrome and nephrosis	12.8 (6.3,19.4)	15.5 (5.6, 25.4)
Influenza and pneumonia	11.6 (4.5, 18.8)	15.1 (8.6, 21.7)
**Good**		
All-cause mortality	1,312.4 (1,225.7, 1,399.2)	1,124.5 (1,033.5, 1,215.5)
Malignant neoplasms	334.2 (286.4, 382.1)	226.8 (192.5, 261.1)
Diseases of heart	331 (288.9, 373.1)	264.5 (221.0, 308.0)
Chronic lower respiratory diseases	54.3 (37.6, 70.9)	76.7 (56.0, 97.4)
Cerebrovascular diseases	67.1 (47.3, 86.9)	62.1 (43.6, 80.7)
Accidents (unintentional injuries)	66.0 (46.3, 85.8)	37.0 (21.3, 52.8)
Diabetes mellitus	48.3 (30.6, 66.0)	39.2 (22.7, 55.6)
Alzheimer's disease	33.4 (22.3, 44.5)	70.7 (51.8, 89.6)
Nephritis, nephrotic syndrome and nephrosis	27.6 (18.6, 36.6)	21.6 (9.2, 33.9)
Influenza and pneumonia	19.1 (9.1, 29.0)	28.3 (15.3, 41.4)
**Fair/Poor**		
All-cause mortality	2,999.4 (2,802.2, 3,196.7)	2,486.7 (2,306.6, 2,666.8)
Malignant neoplasms	644.6 (562.2, 727.1)	416.4 (356.1, 476.7)
Diseases of heart	832.2 (716.2, 948.2)	669.5 (587.2, 751.9)
Chronic lower respiratory diseases	254.3 (194.5, 314.0)	218.8 (170.9, 266.7)
Cerebrovascular diseases	134.1 (99.7, 168.6)	108.5 (78.3, 138.7)
Accidents (unintentional injuries)	55.8 (32.7, 79.0)	61.4 (35.9, 86.9)

© 2026 Francis J et al. *JAMA Network Open*.

Diabetes mellitus	144.1 (102.8, 185.4)	137.8 (100.9, 174.7)
Alzheimer's disease	58.0 (33.2, 82.8)	82.4 (55.2, 109.7)
Nephritis, nephrotic syndrome and nephrosis	68.4 (38.7, 98.1)	71.1 (45.9, 96.3)
Influenza and pneumonia	61.3 (39.7, 82.8)	50.4 (29.3, 71.5)

© 2026 Francis J et al. *JAMA Network Open*.

eTable 9. Adjusted Male-to-Female Hazard Ratios for All-Cause and the Top 9 Causes of Mortality by Self-Rated Health

Cause of Mortality	Fully Adjusted MF HR (95% CI)				*P*-value*
	Excellent	Very Good	Good	Fair/Poor	
All-cause mortality†	1.53 (1.21, 1.75)	1.60 (1.38, 1.76)	1.76 (1.55, 2.00)	1.56 (1.38, 1.76)	0.90
Malignant neoplasms‡	1.22 (0.74, 2.01)	1.64 (1.24, 2.01)	1.83 (1.42, 2.34)	1.75 (1.31, 2.33)	0.89
Diseases of heart§	1.44 (1.01, 2.07)	2.55 (1.80, 3.63)	2.20 (1.79, 2.70)	1.66 (1.33, 2.07)	0.84
Chronic lower respiratory diseases‖	2.13 (0.76, 5.94)	0.79 (0.36, 1.72)	0.88 (0.49, 1.61)	0.98 (0.67, 1.44)	0.56
Cerebrovascular diseases¶	2.07 (0.75, 5.66)	1.45 (0.81, 2.60)	1.87 (1.01, 3.46)	1.80 (1.04, 3.12)	0.97
Accidents (unintentional injuries)**	1.17 (0.33, 4.16)	1.11 (0.63, 1.97)	1.99 (1.08, 3.66)	1.39 (0.62, 3.14)	0.42
Diabetes mellitus††	2.24 (0.0, 9853.20)	2.36 (0.70, 7.99)	1.66 (0.76, 3.62)	1.86 (1.20, 3.07)	0.53
Alzheimer's disease‡‡	0.69 (0.29, 1.63)	1.43 (0.70, 2.92)	0.95 (0.50, 1.79)	1.74 (1.04, 2.92)	0.56
Nephritis, nephrotic syndrome and nephrosis§§	2.02 (0.22, 18.21)	0.92 (0.25, 3.34)	2.34 (0.94, 5.82)	1.26 (0.65, 2.45)	0.71
Influenza and pneumonia¶	0.18 (0.02, 2.02)	1.73 (0.52, 5.69)	1.36 (0.53, 3.48)	2.05 (0.99, 4.26)	0.13

Abbreviations: CI, confidence interval and MF HR, male-to-female hazard ratios

**P*-value from Wald test assessing the statistical significance of the estimated interaction term for sex by self-rated health in the Cox proportional hazards model

†age, age^2, race/ethnicity, education, family income to poverty ratio, health insurance coverage, marital status, routine place to go for healthcare, occupation, smoking intensity, alcohol, body mass index, sedentary behavior, physical activity, history of cardiovascular disease, history of hypertension, history of diabetes, history of cancer

‡age, age^2, race/ethnicity, education, family income to poverty ratio, health insurance coverage, marital status, routine place to go for healthcare, occupation, smoking intensity, alcohol, body mass index, physical activity, history of cancer

§age, age^2, race/ethnicity, education, family income to poverty ratio, health insurance coverage, marital status, routine place to go for healthcare, occupation, smoking intensity, alcohol, body mass index, sedentary behavior, physical activity, history of cardiovascular disease, history of hypertension

‖age, age^2, race/ethnicity, education, family income to poverty ratio, health insurance coverage, marital status, routine place to go for healthcare, occupation, smoking intensity, alcohol, body mass index, sedentary behavior

¶age, age^2, race/ethnicity, education, family income to poverty ratio, health insurance coverage, marital status, routine place to go for healthcare, occupation, smoking intensity, alcohol, body mass index, sedentary behavior, history of cardiovascular disease, history of hypertension

**age, age^2, race/ethnicity, education, family income to poverty ratio, health insurance coverage, marital status, routine place to go for healthcare, occupation, smoking intensity, alcohol, body mass index, , sedentary behavior

††age, age^2, race/ethnicity, education, family income to poverty ratio, health insurance coverage, marital status, routine place to go for healthcare, occupation, smoking intensity, alcohol, body mass index, sedentary behavior, physical activity, history of hypertension, history of diabetes, history of cancer

‡‡age, age^2, race/ethnicity, education, family income to poverty ratio, health insurance coverage, marital status, routine place to go for healthcare, occupation, smoking intensity, alcohol, body mass index, physical activity

§§age, age^2, race/ethnicity, education, family income to poverty ratio, health insurance coverage, marital status, routine place to go for healthcare, occupation, smoking intensity, alcohol, body mass index, physical activity, history of cardiovascular disease, history of hypertension, history of diabetes

© 2026 Francis J et al. *JAMA Network Open*.

eFigure 2. Weighted Male and Female Mortality Rates and Adjusted Male-to-Female Hazard Ratios for All-Cause and the Top 9 Causes of Mortality by Highest Attained Education, Ordered by Highest to Lowest Common Male Mortality Rates
Key: Blue bars represent male mortality rates; red bars represent female mortality rates; boxes indicate male-to-female hazard ratios; whiskers indicate 95% confidence intervals; and dotted line represents the line of unity.

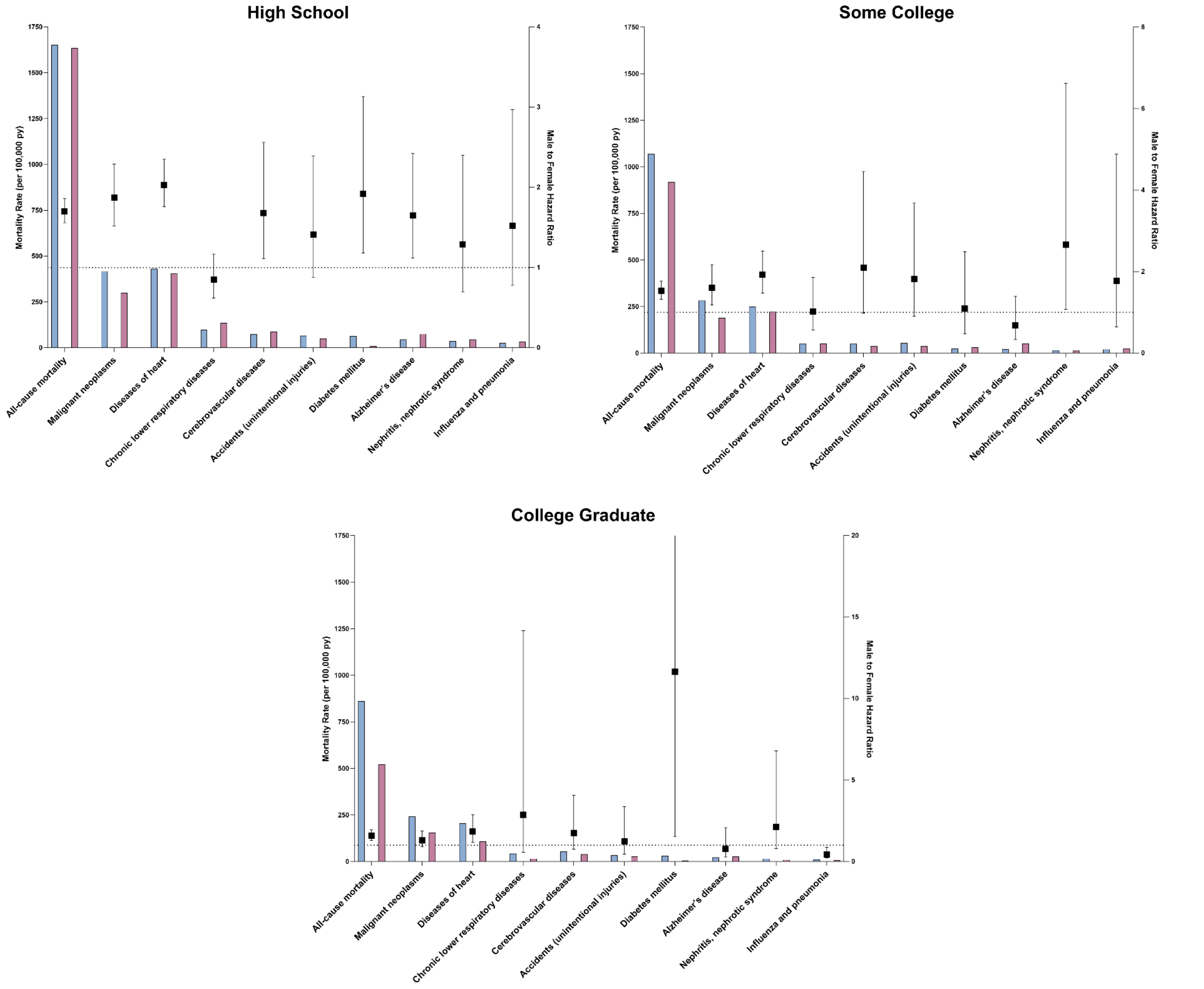

**eTable 10.** Weighted Male and Female Mortality Rates and 95% CIs per 100 000 by Cause of Mortality Within Educational Category		
**Cause of Mortality**	**Male Mortality rate per 100,000 (95% CI)**	**Female Mortality rate per 100,000 (95% CI)**
**High School or Less**		
All-cause mortality	1,651.9 (1,556.1, 1,747.7)	1,636.0 (1,541.7, 1,730.2)
Malignant neoplasms	417.6 (369.7, 465.5)	300.0 (262.2, 337.9)
Diseases of heart	431.0 (389.9, 472.1)	404.8 (363.5, 446.1)
Chronic lower respiratory diseases	99.3 (80.5, 118.1)	136.6 (111.0, 162.2)
Cerebrovascular diseases	75.6 (58.7, 92.5)	88.2 (69.4,107.1)
Accidents (unintentional injuries)	65.7 (46.9, 84.5)	49.9 (33.9, 65.8)
Diabetes mellitus	64.8 (48.7, 80.8)	8.2 (50.7, 83.2)
Alzheimer’s disease	45.4 (33.8, 56.9)	76.0 (59.5, 92.6)
Nephritis, nephrotic syndrome and nephrosis	37.4 (25.4, 49.5)	45.3 (31.7, 58.8)
Influenza and pneumonia	27.1 (17.6, 36.6)	34.3 (21.5, 47.0)
**Some College or Associate Degree**		
All-cause mortality	1,069.0 (977.7, 1,160.4)	918.6 (839.9, 997.3)
Malignant neoplasms	281.7 (238.0, 325.4)	189.6 (154.3, 224.9)
Diseases of heart	248.5 (200.9, 296.0)	221.4 (186.2, 256.6)
Chronic lower respiratory diseases	49.8 (28.3, 71.3)	51.9 (36.8, 67.0)
Cerebrovascular diseases	50.1 (32.9, 67.2)	38.1 (23.9, 52.3)
Accidents (unintentional injuries)	54.9 (37.7, 72.1)	38.0 (19.3, 56.6)
Diabetes mellitus	23.2 (9.8, 36.7)	31.2 (17.5, 44.8)
Alzheimer’s disease	20.4 (9.7, 31.0)	51.4 (34.6, 68.2)
Nephritis, nephrotic syndrome and nephrosis	12.4 (5.0, 19.9)	12.4 (5.6, 19.2)
Influenza and pneumonia	19.6 (10.5, 28.7)	25.4 (14.6, 36.2)
**College Graduate & Above**		
All-cause mortality	861.5 (776.3, 946.8)	521.0 (454.1, 587.9)
Malignant neoplasms	241.0 (193.4, 288.6)	154.6 (117.3, 191.9)
Diseases of heart	205.5 (158.7, 252.4)	108.5 (78.3, 138.8)
Chronic lower respiratory diseases	42.0 (25.1, 58.9)	15.4 (5.0, 25.8)
Cerebrovascular diseases	54.9 (32.9, 76.8)	38.6 (22.4, 54.9)
Accidents (unintentional injuries)	33.9 (17.7, 50.0)	27.1 (9.6, 44.5)
Diabetes mellitus	32.2 (15.0, 49.3)	4.9 (0.2, 9.7)
Alzheimer’s disease	21.7 (12.0, 31.4)	26.4 (13.6, 39.3)
Nephritis, nephrotic syndrome and nephrosis	15.2 (8.1, 22.3)	8.7 (1.5, 15.9)
Influenza and pneumonia	9.2 (1.8, 16.7)	7.5 (2.1, 13.0)

© 2026 Francis J et al. *JAMA Network Open*.

eTable 11. Adjusted Male-to-Female Hazard Ratios for All-Cause and Top 9 Causes of Mortality by Highest Educational Attainment

Cause of Mortality	Fully Adjusted MF HR (95% CI)			*P*-value*
	**High school or less**	**Some college or associate degree**	**College graduate or above**	
All-cause mortality†	1.70 (1.56, 1.86)	1.53 (1.32, 1.77)	1.59 (1.30, 1.94)	0.15
Malignant neoplasms‡	1.87 (1.52, 2.29)	1.60 (1.18, 2.17)	1.30 (0.90, 1.87)	0.33
Diseases of heart§	2.03 (1.76, 2.35)	1.92 (1.47, 2.50)	1.84 (1.19, 2.86)	0.35
Chronic lower respiratory diseases‖	0.85 (0.62, 1.17)	1.02 (0.56, 1.86)	2.87 (0.57, 14.55)	0.05
Cerebrovascular diseases¶	1.68 (1.11, 2.56)	2.09 (0.98, 4.46)	1.75 (0.76, 4.03)	0.88
Accidents (unintentional injuries)**	1.41 (0.84, 2.39)	1.82 (0.90, 3.68)	1.23 (0.45, 3.37)	0.94
Diabetes mellitus††	1.92 (1.18, 3.13)	1.09 (0.47, 2.49)	11.64 (1.54, 87.98)	0.09
Alzheimer's disease‡‡	1.65 (1.12, 2.42)	0.68 (0.33, 1.39)	0.77 (0.28, 2.08)	0.20
Nephritis, nephrotic syndrome and nephrosis§§	1.29 (0.69, 2.39)	2.66 (1.06, 6.69)	2.32 (0.80, 6.79)	0.81
Influenza and pneumonia¶	1.52 (0.78, 2.97)	1.77 (0.64, 4.88)	0.43 (0.21, 0.87)	0.49

Abbreviations: CI, confidence interval and MF HR, male-to-female hazard ratios

**P*-value from Wald test assessing the statistical significance of the estimated interaction term for sex by education in the Cox proportional hazards model

†age, age^2, race/ethnicity, education, family income to poverty ratio, health insurance coverage, marital status, routine place to go for healthcare, occupation, smoking intensity, alcohol, body mass index, sedentary behavior, physical activity, history of cardiovascular disease, history of hypertension, history of diabetes, history of cancer

‡age, age^2, race/ethnicity, education, family income to poverty ratio, health insurance coverage, marital status, routine place to go for healthcare, occupation, smoking intensity, alcohol, body mass index, physical activity, history of cancer

§age, age^2, race/ethnicity, education, family income to poverty ratio, health insurance coverage, marital status, routine place to go for healthcare, occupation, smoking intensity, alcohol, body mass index, sedentary behavior, physical activity, history of cardiovascular disease, history of hypertension

‖age, age^2, race/ethnicity, education, family income to poverty ratio, health insurance coverage, marital status, routine place to go for healthcare, occupation, smoking intensity, alcohol, body mass index, sedentary behavior

¶age, age^2, race/ethnicity, education, family income to poverty ratio, health insurance coverage, marital status, routine place to go for healthcare, occupation, smoking intensity, alcohol, body mass index, sedentary behavior, history of cardiovascular disease, history of hypertension

**age, age^2, race/ethnicity, education, family income to poverty ratio, health insurance coverage, marital status, routine place to go for healthcare, occupation, smoking intensity, alcohol, body mass index, , sedentary behavior

††age, age^2, race/ethnicity, education, family income to poverty ratio, health insurance coverage, marital status, routine place to go for healthcare, occupation, smoking intensity, alcohol, body mass index, sedentary behavior, physical activity, history of hypertension, history of diabetes, history of cancer

‡‡age, age^2, race/ethnicity, education, family income to poverty ratio, health insurance coverage, marital status, routine place to go for healthcare, occupation, smoking intensity, alcohol, body mass index, physical activity

§§age, age^2, race/ethnicity, education, family income to poverty ratio, health insurance coverage, marital status, routine place to go for healthcare, occupation, smoking intensity, alcohol, body mass index, physical activity, history of cardiovascular disease, history of hypertension, history of diabetes

© 2026 Francis J et al. *JAMA Network Open*.
