# Supplementary material for: Sex and All-Cause Mortality in the US, 1999 to 2019
Source: JAMA Netw Open. 2026 Jan 30;9(1):e2556299. doi: 10.1001/jamanetworkopen.2025.56299 (PMC12859720; doi:10.1001/jamanetworkopen.2025.56299)
Supplement: Supplement 2. — Data Sharing Statement [file jamanetwopen-e2556299-s002.pdf]

Data Sharing Statement

Francis. Sex and All-Cause Mortality in the US, 1999 to 2019. *JAMA Netw Open*. Published January 29, 2026. doi:10.1001/jamanetworkopen.2025.56299

Data
Data available: Yes
Data types: Data dictionary
How to access data: https://wwwn.cdc.gov/nchs/nhanes/default.aspx These are publicly available datasets.
When available: With publication

Supporting Documents
Document types: None

Additional Information
Who can access the data: These are publicly available data.
Types of analyses: N/A
Mechanisms of data availability: N/A
